# Coupling Photoresponsive Transmembrane Ion Transport with Transition Metal Catalysis

**DOI:** 10.1021/jacs.3c13801

**Published:** 2024-02-09

**Authors:** Xiangyu Chao, Toby G. Johnson, Maria-Carmen Temian, Andrew Docker, Antoine L. D. Wallabregue, Aaron Scott, Stuart J. Conway, Matthew J. Langton

**Affiliations:** † Chemistry Research Laboratory, University of Oxford, Mansfield Road, Oxford OX1 3TA, U.K.; ‡ Department of Chemistry & Biochemistry, University of California Los Angeles, 607 Charles E. Young Drive East, P.O. Box 951569, Los Angeles, California 90095-1569, United States

## Abstract

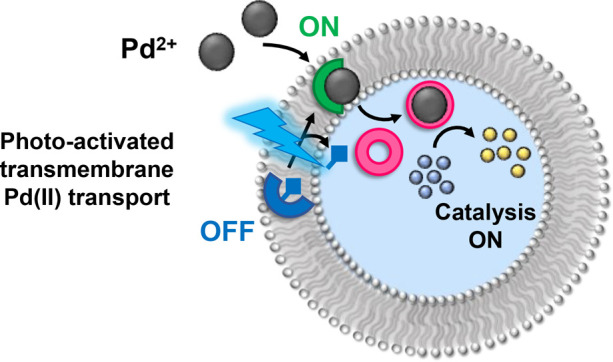

Artificial ion transporters have been explored both as tools for studying fundamental ion transport processes and as potential therapeutics for cancer and channelopathies. Here we demonstrate that synthetic transporters may also be used to regulate the transport of catalytic metal ions across lipid membranes and thus control chemical reactivity inside lipid-bound compartments. We show that acyclic lipophilic pyridyltriazoles enable Pd(II) cations to be transported from the external aqueous phase across the lipid bilayer and into the interior of large unilamellar vesicles. *In situ* reduction generates Pd(0) species, which catalyze the generation of a fluorescent product. Photocaging the Pd(II) transporter allows for photoactivation of the transport process and hence photocontrol over the internal catalysis process. This work demonstrates that artificial transporters enable control over catalysis inside artificial cell-like systems, which could form the basis of biocompatible nanoreactors for applications such as drug synthesis and delivery or to mediate phototargeted catalyst delivery into cells.

In nature, lipid bilayers form compartments essential for life, decoupling the chemical environments on either side of the membrane. Studying artificial chemical reactivity within lipid bilayer membranes continues to attract much interest in the context of membrane-anchored catalysts,^1−4^ artificial photosynthesis,^5−7^ self-replicating vesicles,^8^ catalytic pores,^9^ and signal transduction systems.^10−12^ Controlling chemical reactions within living cells is an area of significant current research, with applications ranging from in-cell decaging/activation of fluorophores, drugs, and proteins to in-cell synthesis of biologically active molecules and protein labeling.^13−16^ Artificial membrane-bound compartments are advantageous for catalysis: they provide confined nanoscale reaction vessels in which the membrane separates chemically incompatible processes, and the low-dielectric environment within the membrane typically strengthens non-covalent interactions and solubilizes lipophilic organic molecules. Furthermore, compartmentalization suggests future opportunities in mediating multistep chemical transformations, each operating in a chemically insulated and controlled compartment. This concept has been demonstrated using biological protein systems.^17−21^ However, controlling abiotic chemical reactions such as transition-metal-mediated catalysis inside membrane-bound compartments presents a significant challenge. Membranes are typically impermeable to polar molecules and catalytically active ions, and therefore, a controlled and selective transmembrane transport process is required to access the interior.

Artificial anion transporters are now very well established,^22−24^ and stimuli-responsive systems that enable temporal control over activity are emerging.^25,26^ Cation transporters have received comparably less interest in recent years, with focus primarily on alkali metal cations, and only a handful of ionophores for copper and zinc ions have been reported.^27−31^ Recently, Matile and co-workers reported a combined transport–catalysis system in which pnictogen-bonding anion transporters were used as Lewis acidic catalysts promoting the formation of oligoepoxide sodium transporters within lipid membranes.^32^ However, to the best of our knowledge, combining synthetic cation transporters with transition metal catalysis is unprecedented.

Herein we demonstrate this concept by developing the first example of an artificial ion transporter for Pd(II) cations whereby the transmembrane transport of Pd(II) into the lumen of lipid bilayer vesicles, followed by *in situ* reduction to Pd(0), triggers catalysis. We show that the transport process, and hence intravesicle catalysis, can be controlled by light through photocaging the transporter (Figure 1).

**Figure 1:**
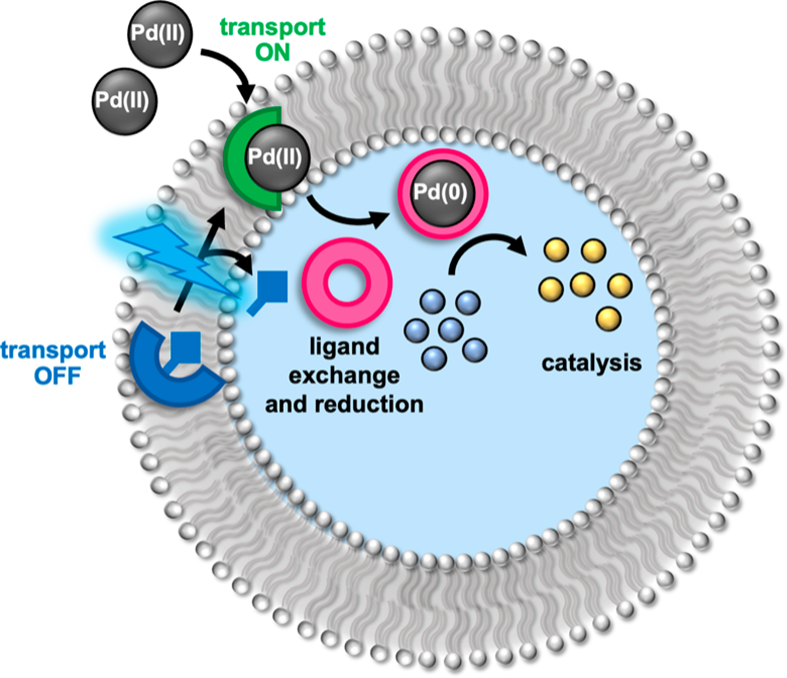
Schematic representation of phototriggered transport-coupled catalysis within a lipid bound compartment. *In situ* photodecaging of a procarrier (blue) generates a mobile ion carrier (green) for Pd(II), which facilitates transmembrane Pd transport. Ligand exchange with a water-soluble phosphine ligand (pink) generates a Pd(0) species within the internal aqueous phase and switches on catalysis of encapsulated substrate molecules.

We sought to target the transport of palladium ions to demonstrate photocontrolled, transport-mediated intracompartment catalysis, given the extensive Pd-mediated coupling chemistry available, particularly in aqueous solution^33^ as well as in living cells.^34^ Palladium has been utilized for in-cell drug molecule synthesis,^35^ cell-surface labeling,^36^ and decaging of bioactive (macro)molecules.^37,38^ Cellular uptake of Pd(II) has been achieved using peptide–Pd complexes^39^ and that of Pd(0) using nanoparticle “Trojan horse” delivery systems,^40−42^ but their permeability (and hence catalytic activity) cannot be controlled in response to external stimuli. This is required for targeted activation applications within artificial cells or living cells.

We first explored the possibility of transporting Pd(II) cations using synthetic transporters in large unilamellar vesicles (LUVs). To this end, we targeted a series of pyridyl 1,2,3-triazole-based ligands of varying denticities and donor atom arrangements appended with lipophilic alkyl chains as potentially suitable ionophores for Pd(II) binding and membrane transport.^43,44^ We have previously used triazole derivatives, which are readily accessible via CuAAC click chemistry, as anion transporters, in which maximum transport activity occurs at log *P* of 5–6.^45,46^ Accordingly, the bidentate pyridyltriazole transporter **1** and bistriazole analogues **2**(47) and **3**(48) (Figure 2), with clog*P* values in this range (5.8, 5.8, and 6.3, respectively), were prepared. Full synthetic procedures and characterization are available in the Supporting Information. To detect the Pd transport, we prepared non-fluorescent water-soluble, membrane-impermeable sensor **4**. A deallylation reaction, which necessarily proceeds through a Pd(0)–phosphine active species generated by *in situ* reduction,^49^ affords the fluorophore 8-hydroxypyrene-1,3,6-trisulfonate (**5**, HPTS) in a similar manner to previously reported membrane-permeable probes.^40,50,51^

**Figure 2:**
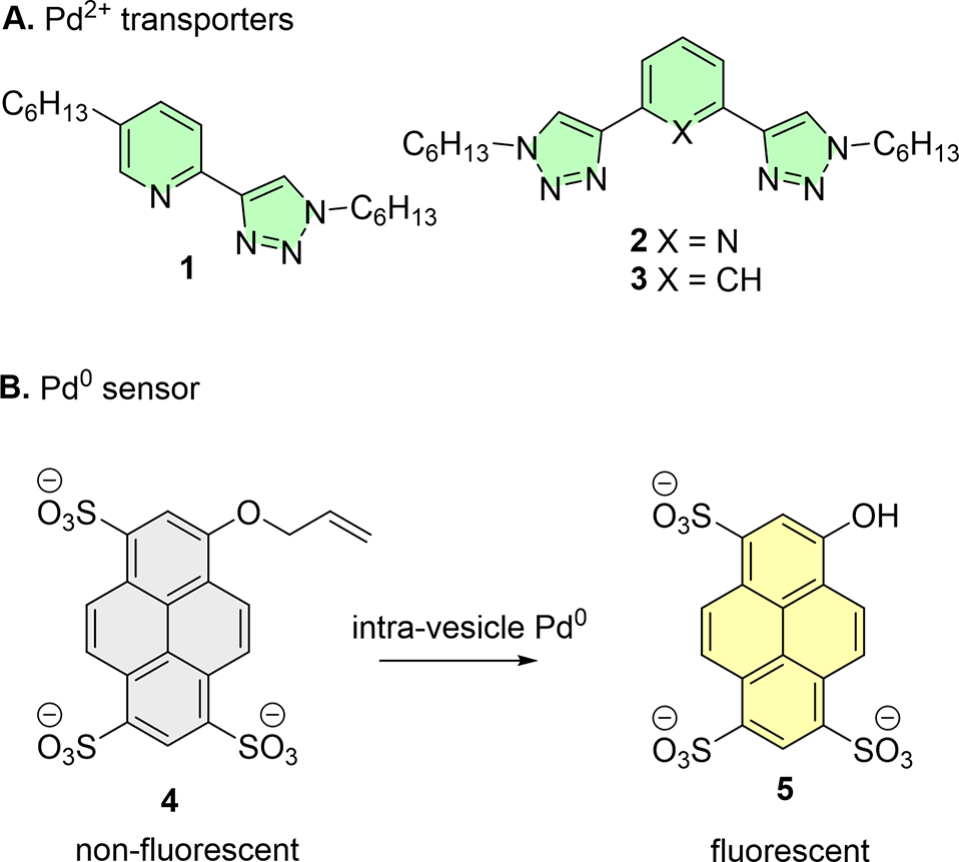
(A) Pd transporters **1**–**3**. (B) Pd(0)-mediated allyl deprotection of **4** to form HPTS **5**.

The palladium(II) cation transport-coupled catalysis activities of pyridyltriazole derivatives **1**–**3** were determined in 1-palmitoyl-2-oleoyl-*sn-*glycero-3-phosphocholine large unilamellar vesicles (POPC LUVs) (lipid concentration 100 μM) loaded with 1 mM **4** and 2 mM water-soluble phosphine 3,3′,3″-phosphanetriyltris(benzenesulfonic acid) trisodium salt (TPPTS) in 100 mM NaNO_3_ aqueous solution buffered to pH 7.0 with 10 mM HEPES. The excess phosphine ligand is present to coordinate the Pd(II) delivered to the internal aqueous phase to facilitate *in situ* reduction to Pd(0) for the deallylation of **4**(37,52) and stabilize the formed Pd^0^(TPPTS)_*n*_ complex to inhibit palladium nanoparticle or palladium black formation.^53^ A gradient of Pd(II) ions was applied by addition of 100 μM Pd(NO_3_)_2_ to the external aqueous phase of the LUV suspension, followed by addition of the carrier as a DMSO solution (<0.5% v/v). The generation of fluorescent product **5** from **4** was monitored using fluorescence spectroscopy. Following the addition of Pd(NO_3_)_2_ to the LUVs, no generation of **5** was observed in the absence of transporter **1**, revealing the negligible membrane permeability of the Pd(II) salt (Figure 3A, black line). In the presence of **1**, however, rapid generation of **5** was observed, consistent with the delivery and *in situ* reduction of Pd(II) to catalytically active Pd(0) species inside the vesicles (Figure 3A), the rate of which was enhanced with increasing membrane loading of **1**. The intravesicle reaction of **4** to form **5** was complete after approximately 20 min in the presence of 1 mol % **1**, which was confirmed by the lack of rate enhancement upon addition of excess Pd(NO_3_)_2_ at the end of the experiment (Figure S18). Assuming complete dissipation of the 100 μM Pd^2+^ gradient by **1**, and with an internal substrate concentration of 1 mM, this equates to each Pd species catalyzing the deallylation of 10 substrate molecules (i.e., catalytic turnover is achieved in the vesicle interior). The observed activity corresponds to a combined transport–catalysis mechanism, the transport step of which is presumably dominated by the diffusion of the neutral **1**·Pd(NO_3_)_2_ complex, given that Pd(NO_3_)_2_ is present in 100-fold excess compared to **1**, in 100 mM NaNO_3_ solution. Calcein release assays and dynamic light scattering (DLS) experiments confirmed that the LUV membrane integrity was maintained during these experiments (Figure S26 and Table S1).

Analysis of the dependence of the fractional activities (*I*_rel_ at 1000 s; Figure 3A) afforded an effective concentration value required to reach 50% activity (EC_50_) of ∼0.25 mol % **1** (with respect to lipid). The bistriazole derivative **2** was inactive (Figure 3B), and **3** exhibited poor activity, with an EC_50_ value too low to be determined (>10 mol %). We postulate that the affinity of these tridentate ligands exceeds the optimum stability range for transport, which is itself a balance between adequate affinity of the carrier for the ion at the interface and sufficient coordination lability to allow for ion release (a so-called “Goldilocks” effect^55^).

A lack of catalytic activity in the presence of transporter **1** but in the absence of internal TPPTS revealed the requirement for competitive phosphine–Pd(II) coordination in the internal aqueous phase and *in situ* reduction to Pd(0) (Figure S21). This is in agreement with previous studies on the formation of Pd(0)–TPPTS complexes from Pd(II) precursors in aqueous solution.^56^ The requirement for a Pd(0) species for the deallylation of **4** was also confirmed by additional control experiments in which lipophilic palladium sources Pd^0^(PPh_3_)_4_ and Pd^II^(PhCN)_2_Cl_2_ were added to LUVs in the absence of the reducing agent TPPTS (Figure S22). Catalytic deallylation of **4** was only observed with the Pd(0)–phosphine complex and not with the Pd(II) species. We also explored the effect of varying the concentration of TPPTS inside the vesicles, given that the putative catalytically active Pd(0)–phosphine complex would presumably be deactivated if coordinatively saturated.^56,57^ Indeed, increasing the concentration of TPPTS inside the LUVs to 4 mM diminished the reaction rate (Figure S23), consistent with saturation of the Pd coordination sphere and in line with results in aqueous solution (Figure S24). Optimum activity within LUVs was achieved at 2 mM TPPTS loading of the vesicles in the presence of a 100 μM Pd^2+^ gradient.

A challenge in transition metal catalysis in cellular systems is the current lack of the ability to target and activate catalysis with spatial and/or temporal control. To address this, we sought to develop a photocaged analog of **1** that could be activated with light in order to trigger transmembrane Pd transport and catalysis in LUVs. To this end, we prepared photocaged procarrier **6**, in which the pyridine motif of carrier **1** is alkylated with a red-shifted coumarin derivative to prevent the binding and transport of Pd(II) (Figure 4A). Such derivatives have previously been shown to be effective photocages for pyridines.^58^ Carrier **1** could be readily generated from procarrier **6** by irradiation of a DMSO solution at 455 nm using an ∼1 W LED, as determined by ^1^H NMR experiments (Figures S25 and S26).

To explore light-activated transport-coupled catalysis in LUVs, we studied the activity of **1** generated from **6** by photoirradiation. The caged derivative **6** was inactive in the Pd(II) transport experiments due to blocking of the pyridine Lewis basic donor atom (Figure 4B, blue data). In contrast, following *ex situ* photoirradiation of **6** in DMSO solution at 455 nm to quantitatively generate **1** and subsequent addition of the photocleaved products to the LUVs, transport–catalysis activity comparable to that of an equivalent concentration of **1** was achieved (green data). Pleasingly, *in situ* photoactivation, in which **1** is generated from **6** already incorporated into the membrane of the LUVs, could be achieved by directly irradiating vesicles containing **6** prior to addition of the Pd(II) ion gradient (Figure 4C). Time-dependent activation studies demonstrated that comparable activity to *ex situ* activation, or that of an equivalent concentration of **1**, was achieved following 5 s of irradiation of the cuvette with 455 nm light. Overall these results demonstrate that the transporter could be efficiently generated in the membrane by photodecaging, which in turn provides a mechanism by which to photoactivate Pd-mediated catalysis inside vesicles.

**Figure 3:**
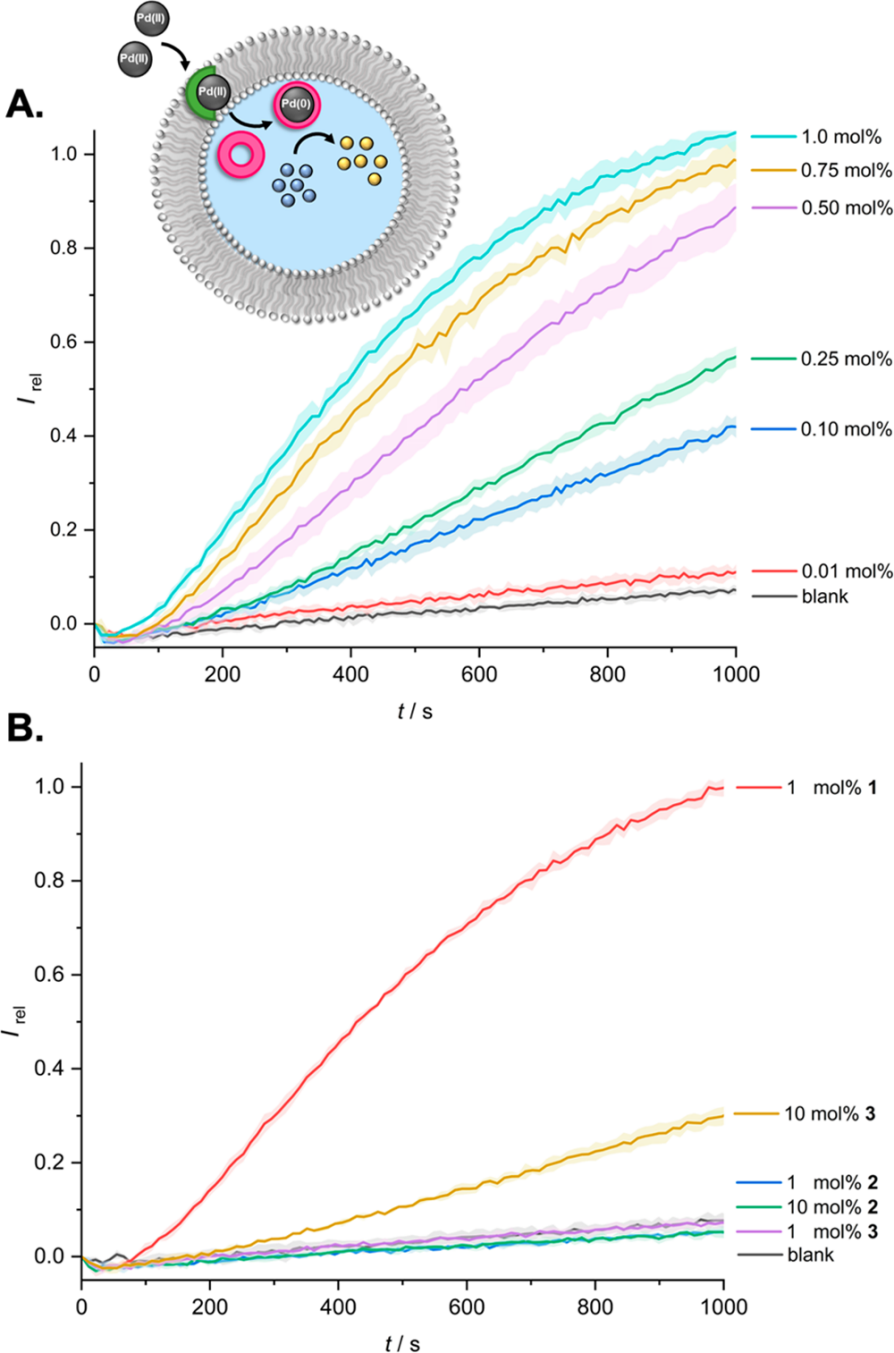
Time dependence of the normalized fluorescence emission intensity at 510 nm (exciting at 460 nm) due to Pd-catalyzed deallylation of **4** in POPC LUVs triggered by transmembrane Pd^2+^ transport. Experiments were conducted in 200 nm POPC LUVs (100 μM lipid) containing 1 mM allyl-HPTS **4**, 2 mM TPPTS, and 100 mM NaNO_3_ in 10 mM HEPES buffer at pH 7.0. A Pd^2+^ ion gradient was generated by addition of 100 μM Pd(NO_3_)_2_(aq) at *t* = 0 s. (A) Concentration dependence of **1** on intravesicle catalytic activity (mol % with respect to lipid). Blank refers to data in the presence of Pd^2+^ but in the absence of **1**. (B) Data for transporters **2** and **3** (10 mol %) in comparison with **1** (1 mol %). Data were normalized to the activity of **1** (1 mol % at 1000 s). Shaded regions represent standard deviations of three repeats.

**Figure 4:**
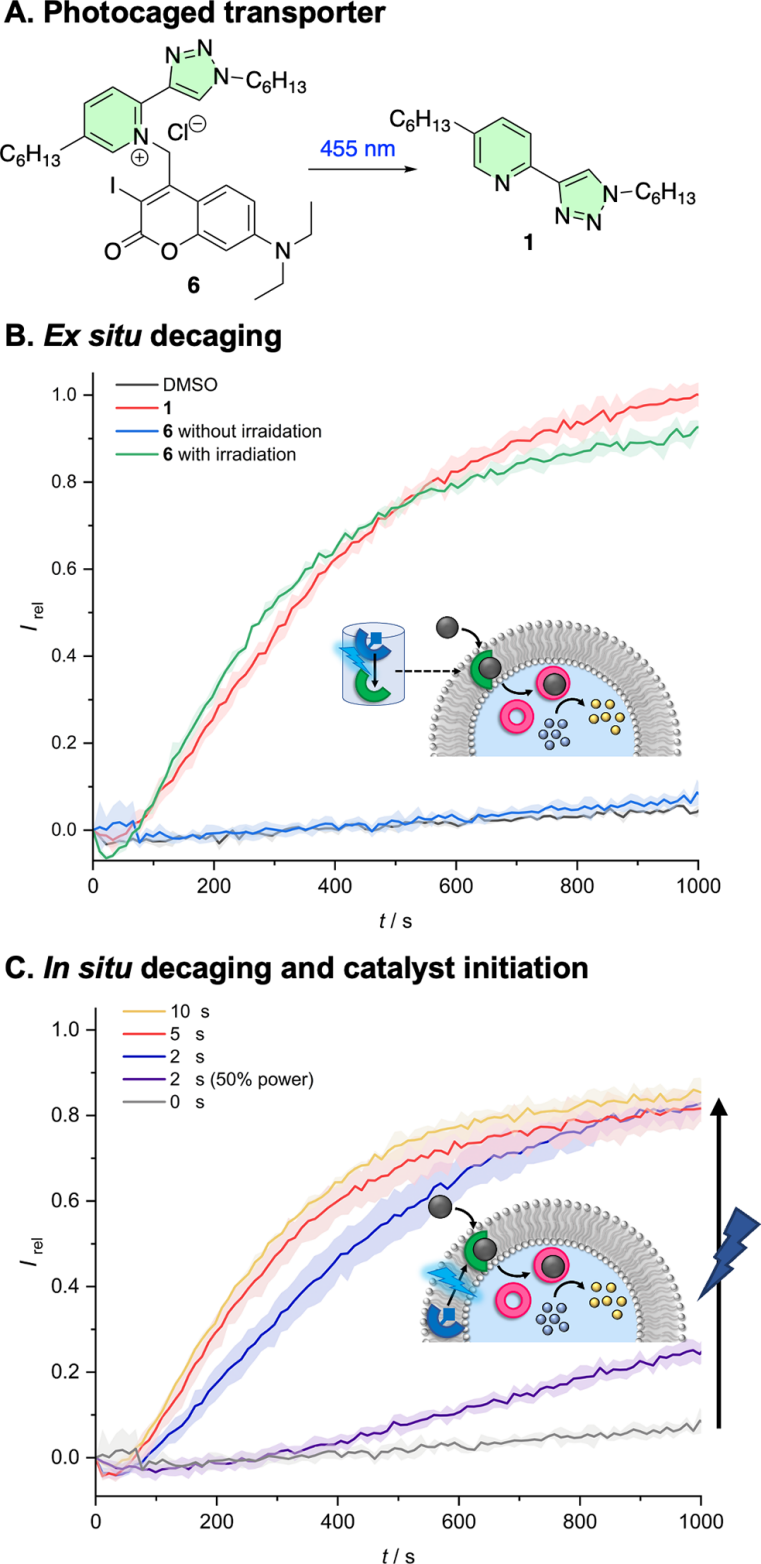
Light-activated Pd transport and catalysis. (A) Photocaged procarrier **6**. (B) Intravesicle Pd catalysis data for procarrier **6** (blue data) and after photodecaging (green), compared with the activity of **1** (red). (C) *In situ* switch-on activation triggered by photogeneration of **1** from **6** in the membrane of LUVs after the stated irradiation time with an ∼1 W 455 nm LED. Experimental conditions as in Figure 3.

In summary, we report the first example of a synthetic transport system capable of the light-activated transport of catalytically active transition metal ions to trigger intravesicle catalysis. Lipophilic pyridyltriazole mobile ion carriers are capable of extracting Pd(II) cations from the external aqueous phase, crossing the membrane, and exchanging with encapsulated phosphine ligands to generate a catalytically active Pd(0)–phosphine species able to mediate a deallylation reaction. The activity of the intra-vesicle catalysis is dependent on the carrier concentration in the membrane and carrier coordination properties. By photocaging the Pd carrier, a light-activated Pd transport–catalysis system was engineered, which enabled phototriggered catalysis inside vesicles by regulating the delivery of Pd(II) across the boundary lipid bilayer membrane. These results demonstrate the potential of synthetic transport systems to deliver catalytic cations across cell membranes, and work toward this goal is ongoing in our laboratories.

## Supporting Information Available

Additional experimental details, synthesis and characterization, photophysical characterization, and transport and catalysis experiments (PDF)
