# Supplementary material for: Coupling Photoresponsive Transmembrane Ion Transport with Transition Metal Catalysis
Source: J Am Chem Soc. 2024 Feb 9;146(7):4351–6. doi: 10.1021/jacs.3c13801 (PMC10885138; doi:10.1021/jacs.3c13801)
Supplement: Supplementary file 1 — ja3c13801_si_001.pdf [file ja3c13801_si_001.pdf]

Supporting Information for:

Coupling photo-responsive transmembrane ion transport with transition metal catalysis

Xiangyu Chao[a]†, Toby G. Johnson[a]†, Maria-Carmen Temian[a], Andrew Docker[a], Antoine L. D. Wallabregue[a], Aaron Scott[a], Stuart J. Conway[a,b] and Matthew J. Langton[a]*

[a]Chemistry Research Laboratory, University of Oxford, Mansfield Road, Oxford, OX1 3TA, UK

[b]Department of Chemistry & Biochemistry, University of California Los Angeles, 607 Charles E. Young Drive East, P.O. Box 951569, Los Angeles, California 90095-1569, USA

Contents

1 Materials and methods 2
2 Synthesis and characterization 3
3 UV-Visible absorption analysis 14
4 Pd(II) transport experiments 16
5 Pd(II) catalysis experiments in solution 20
6 Photo-deprotection experiments 21
7 Irradiation studies on Pd(II) transport assays 22
8 Calcein leakage assays 22
9 Dynamic light scattering 24
10 References 25

1 Materials and methods

All reagents and solvents were purchased from commercial sources and used without further purification. Lipids were purchased from Avanti polar lipids and used without further purification. Where necessary, solvents were dried by passing through an MBraun MPSP-800 column and degassed with nitrogen. Triethylamine was distilled from and stored over potassium hydroxide. Normal phase silica gel flash column chromatography was performed manually using Merck® silica gel 60 under a positive pressure of nitrogen. Where mixtures of solvents were used, ratios are reported by volume. NMR spectra were recorded on a Bruker AVIII 400, Bruker AVII 500 (with cryoprobe) and Bruker AVIII 500 spectrometers. Chemical shifts are reported as δ values in ppm. Mass spectra were carried out on a Waters Micromass LCT and Bruker microTOF spectrometers. DLS analysis was performed on Malvern Zetasizer Nano with a 532 nm laser as the light source. Fluorescence spectroscopic data were recorded using an Agilent Cary Eclipse or Horiba Duetta fluorescence spectrophotometer, equipped with a Peltier temperature controller and stirrer. Experiments were conducted at 25 °C unless otherwise stated. Vesicles were prepared as described below using Avestin "LiposoFast" extruder apparatus, equipped with polycarbonate membranes with 200 nm pores. GPC purification of vesicles was carried out using GE Healthcare PD-10 desalting columns prepacked with Sephadex G 25 medium.

Abbreviations

DIPEA: *N,N*-Diisopropylethylamine; **EDTA:** Ethylenediaminetetraacetic acid; **HEPES**: *N*-(2-Hydroxyethyl)piperazine-*N*'-(2-ethanesulfonic acid); **HPTS**: 8-Hydroxy-1,3,6-pyrenetrisulfonate; **HRMS**: High resolution mass spectrometry; **LUVs**: Large unilamellar vesicles; **MW:** Microwave irradiation; **NIS**: *N*-Iodosuccinimide; **POPC**: 1-Palmitoyl-2-oleoyl-*sn*-glycero-3-phosphocholine; **RT**: Room temperature; **TMS**: Trimethylsilane; **TPPTS:** 3,3′,3′′-Phosphanetriyltris(benzenesulfonic acid) trisodium salt.

2 Synthesis and characterization

2.1 General comments.

Compounds **2**[1], **3**[2], **4**[3], **S2**[4], **S3**[5], **S8**[6], **S9**[7], **S10**[7] were prepared according to literature procedures.

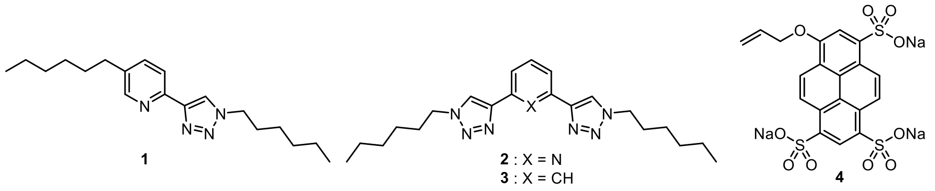

***Figure S1**. Pd(II) Transporters (**1-3**) and Pd(0) responsive probe (**4**) used in this study.*

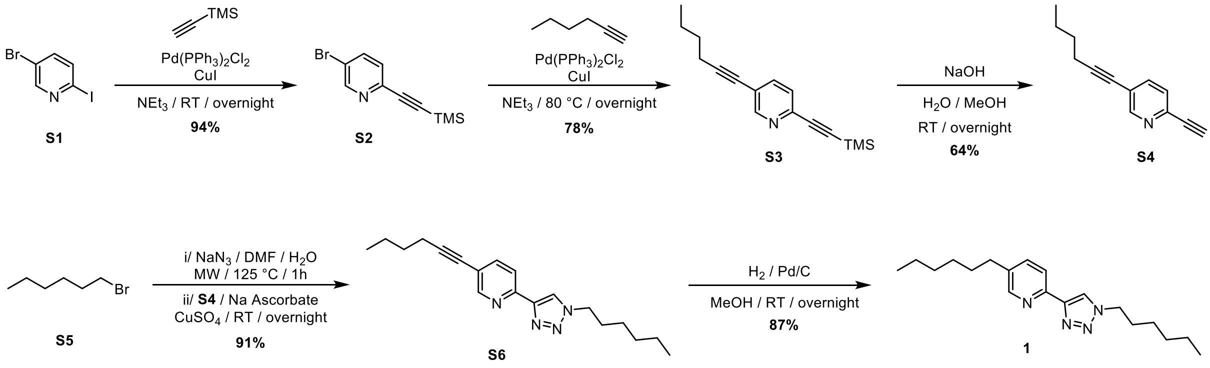

***Scheme S1.** Synthesis of mobile carrier **1**.*

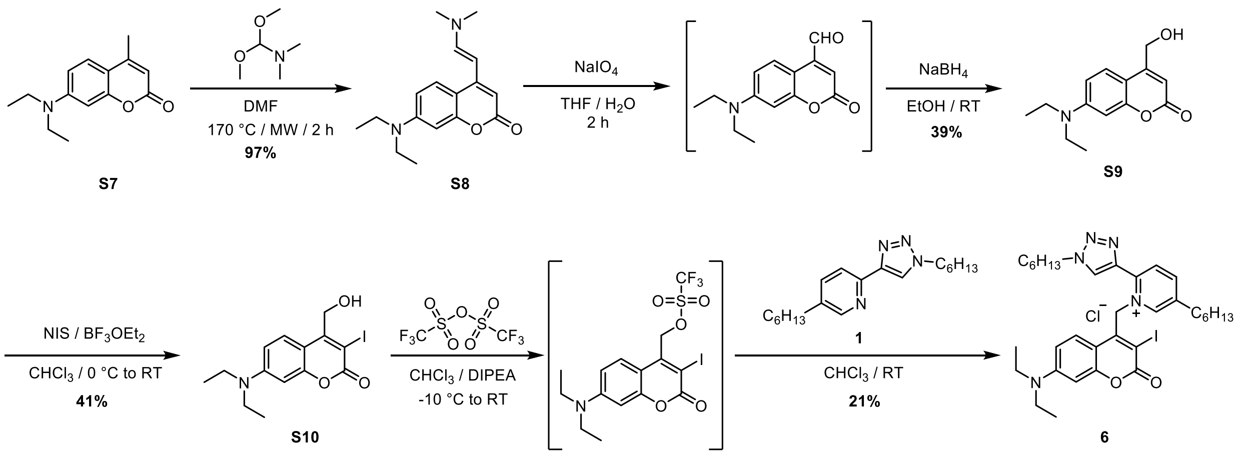

***Scheme S2.** Synthesis of photo-caged mobile carrier **6**.*

2.2 Allyl-HPTS trisodium salt 4.

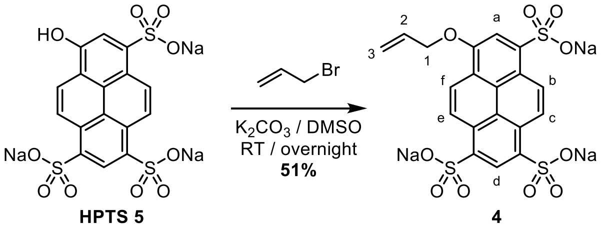

This known compound was prepared *via* a novel procedure: 8-Hydroxypyrene-1,3,6-trisulfonic acid trisodium salt (200 mg, 0.4 mmol, 1 Equiv.), allyl bromide (70 µL, 0.8 mmol, 2 Equiv.) and K_2CO_3 (530 mg, 3.8 mmol, 10 Equiv.) were dissolved in DMSO (3 mL) and left to stir at RT overnight. Addition of acetone (10 mL) to the reaction mixture generated an orange precipitate which was isolated by filtration. This material was then washed with excess acetone and dried to afford the title compound as a yellow-orange solid (110 mg, 0.2 mmol, 51%). The characterisation matches that reported in the literature.[3]

^{1}H NMR (400 MHz, D_2O) δ 9.18 (s, $1H_d$), 9.15 (d, *J* = 9.8 Hz, 1H), 9.05 (d, *J* = 9.7 Hz, 1H), 8.98 (d, *J* = 9.8 Hz, 1H), 8.88 (d, *J* = 9.6 Hz, 1H), 8.40 (s, $1H_a$), 6.38 – 6.27 (m, $1H_2$), 5.64 (d, *J* = 17.3 Hz, $1H_3$), 5.46 (d, *J* = 10.6 Hz, $1H_3$), 5.12 (s, $2H_1$).

2.3 5-bromo-2-((trimethylsilyl)ethynyl)pyridine S2.

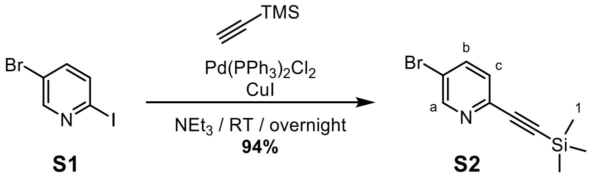

This known compound was prepared *via* a novel procedure: To a mixture of 5-bromo-2-iodopyridine **S1** (2.0 g, 4.9 mmol, 1 Equiv.), bis(triphenylphosphine)palladium(II) dichloride (300 mg, 0.4 mmol, 0.06 Equiv.), copper(I) iodide (82 mg, 0.4 mmol, 0.06 Equiv.) under N_2 was added a degassed solution of ethynyltrimethylsilane (692 mg, 7.0 mmol, 1 Equiv.) in NEt_3 (25 mL). The reaction mixture was left to stir at RT overnight, then diluted with H_2O (100 mL) and extracted with EtOAc (3 × 100 mL), the combined organic extracts were washed with 0.1 M NH_4OH / EDTA $_{(aq)}$ (1 × 25 mL), H_2O (3 × 100 mL) and dried ($MgSO_4$), filtered and concentrated to give a crude material. This crude material was purified by silica gel flash chromatography (10% EtOAc / hexane) to afford the title compound as a white solid (1.68 g, 6.6 mmol, 94%). The characterisation matches that reported in the literature.[4]

^{1}H NMR (400 MHz, $CDCl_3$) δ 8.62 (dd, *J* = 2.3, 0.8 Hz, $1H_a$), 7.77 (dd, *J* = 8.3, 2.4 Hz, $1H_b$), 7.34 (dd, *J* = 8.4, 0.8 Hz, $1H_c$), 0.27 (s, $9H_1$).

2.4 5-(hex-1-yn-1-yl)-2-((trimethylsilyl)ethynyl)pyridine S3.

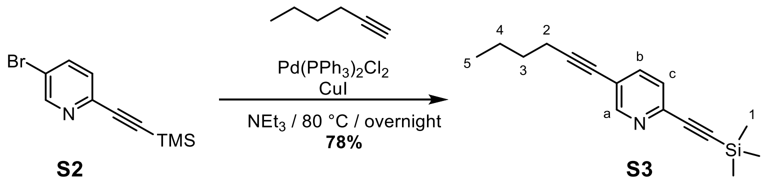

This known compound was prepared *via* a novel procedure: To a mixture 5-bromo-2-((trimethylsilyl)ethynyl)pyridine **S2** (200 mg, 0.79 mmol, 1 Equiv.), bis(triphenylphosphine)palladium(II) dichloride (10.5 mg, 0.06 mmol, 0.07 Equiv.), copper(I) iodide (39 mg, 0.06 mmol, 0.07 Equiv.) under N_2 was added a degassed solution of 1-hexyne (0.18 mL, 1.6 mmol, 2 Equiv.) in NEt_3 (8 mL). The reaction mixture was left to stir at 80 °C overnight, then diluted with H_2O (25 mL) and extracted with CH_2Cl_2 (3 × 25 mL), the combined organic extracts were washed 0.1 M NH_4OH / EDTA $_{(aq)}$ (1 × 25 mL), H_2O (2 × 25 mL) and brine (25 mL) and dried ($MgSO_4$), filtered and concentrated to give a crude material. This crude material was purified by silica gel flash chromatography (10% EtOAc / hexane) to afford the title compound (157 mg, 0.6 mmol, 78%). The characterisation matches that reported in the literature.[5]

^{1}H NMR (600 MHz, $CDCl_3$) δ 8.56 (dd, *J* = 2.2, 0.9 Hz, $1H_a$), 7.61 (dd, *J* = 8.1, 2.1 Hz, $1H_b$), 7.36 (dd, *J* = 8.1, 0.9 Hz, $1H_c$), 2.43 (t, *J* = 7.1 Hz, $2H_2$), 1.63 – 1.55 (m, $2H_3$), 1.52 – 1.42 (m, $2H_4$), 0.95 (t, *J* = 7.3 Hz, $3H_5$), 0.26 (s, $9H_1$)

2.5 2-ethynyl-5-(hex-1-yn-1-yl)pyridine S4.

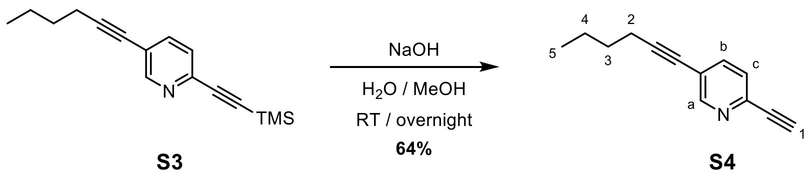

5-(hex-1-yn-1-yl)-2-((trimethylsilyl)ethynyl)pyridine **S3** (157 mg, 0.61 mmol, 1 Equiv.) was dissolved in a solution of MeOH (3.6 mL) and 1M NaOH solution (1.2 mL). The reaction mixture was left to stir at RT overnight, then diluted with H_2O (25 mL) and extracted with EtOAc (3 × 25 mL), the combined organic extracts were washed with H_2O (25 mL) and brine (25 mL) and dried ($MgSO_4$), filtered and concentrated to give a crude material. This crude material was purified by silica gel flash chromatography (10% EtOAc / hexane) to afford the title compound (72 mg, 0.4 mmol, 64%).

^{1}H NMR (400 MHz, $CDCl_3$) δ 8.57 (d, *J* = 1.6 Hz, $1H_a$), 7.63 (dd, *J* = 8.1, 2.1 Hz, $1H_b$), 7.39 (d, *J* = 8.1 Hz, $1H_c$), 3.22 (s, $1H_1$), 2.44 (t, *J* = 7.1 Hz, $2H_2$), 1.64 – 1.56 (m, $2H_3$), 1.53 – 1.41 (m, $2H_4$), 0.95 (t, *J* = 7.3 Hz, $3H_5$).

^{13}C NMR (151 MHz, $CDCl_3$) δ 152.7, 140.3, 138.6, 126.8, 121.2, 96.4, 82.7, 78.5, 77.3, 30.7, 22.2, 19.4, 13.7.

HRMS-ESI (m/z) Calculated for $C_{13}H_{14}N$ $[M+H]^+$, 184.1121; found 184.1118.

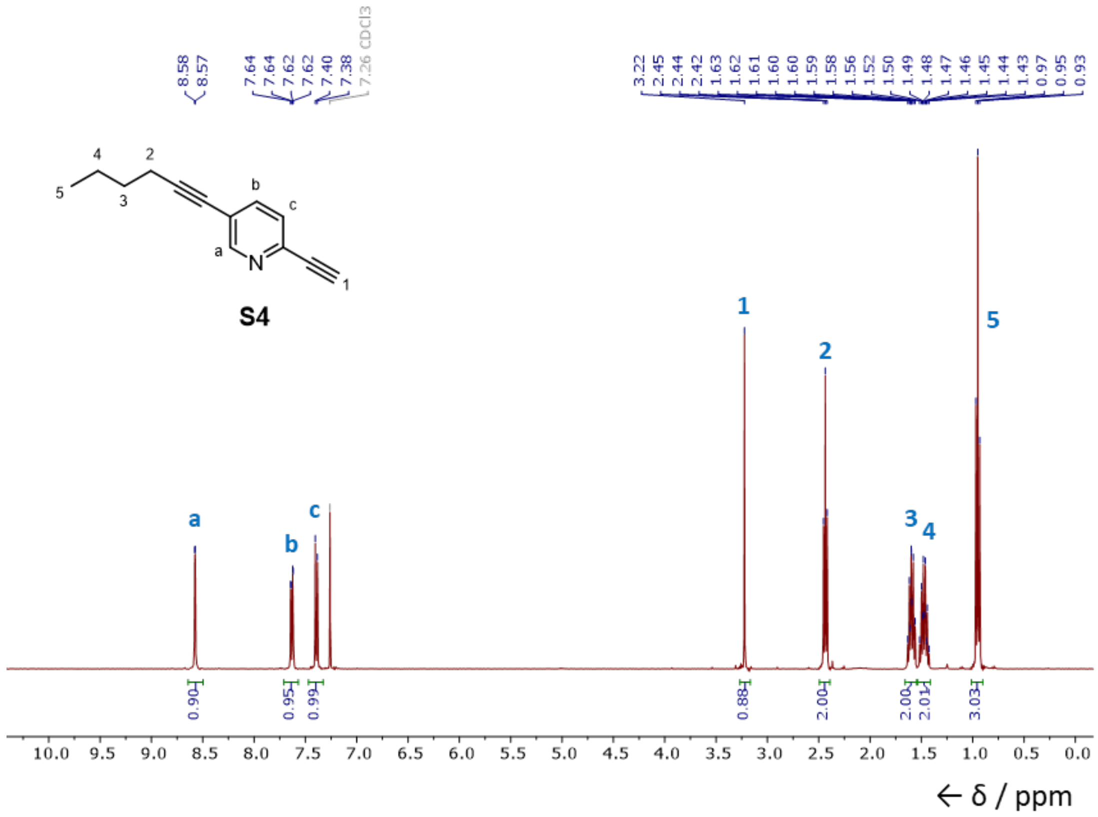

__Figure S2.__ ^{1}H NMR spectrum of compound __S4__ (CDCl$_3$, 600 MHz, 298 K).

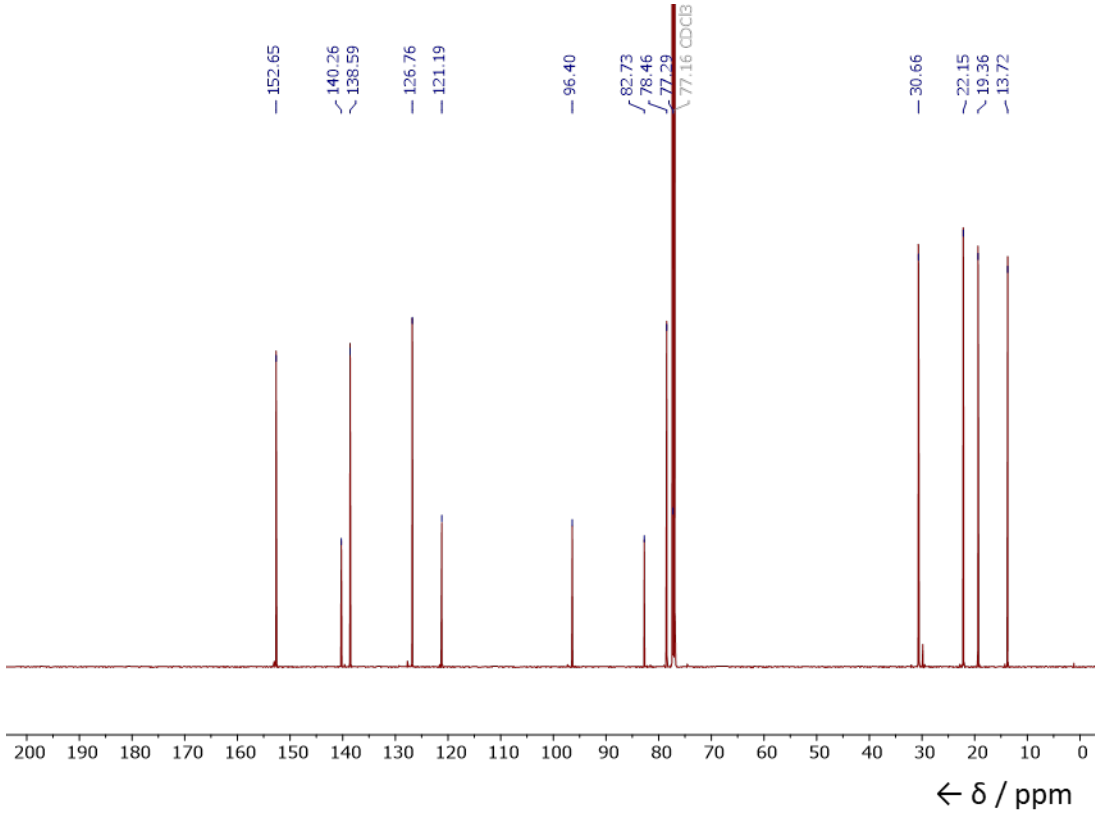

__Figure S3.__ ^{13}C NMR spectrum of compound __S4__ (CDCl$_3$, 150 MHz, 298 K).

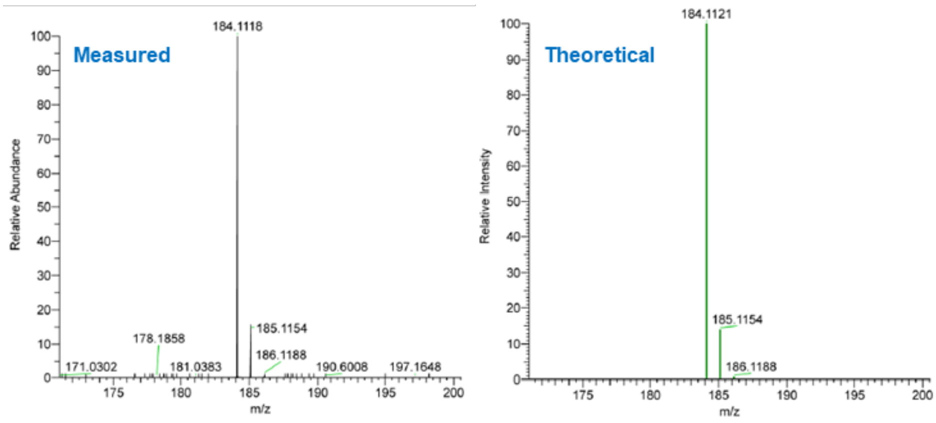

Figure S4. *HRMS spectrum of compound* ***S4****. Calculated for $C_{13}H_{14}N$ [M+H]$^+$, 184.1121; found 184.1118.*

2.6 5-(hex-1-yn-1-yl)-2-(1-hexyl-1H-1,2,3-triazol-4-yl)pyridine S6.

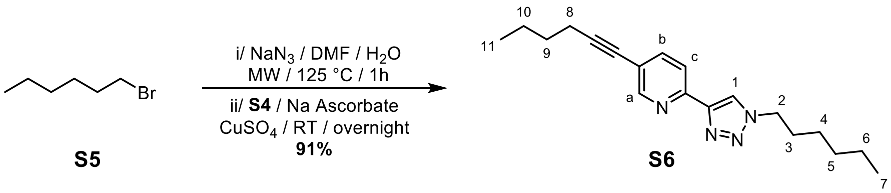

1-Bromohexane (59 mg, 0.35 mmol, 1 Equiv.) and sodium azide (23 mg, 0.35 mmol, 1 Equiv.) were dissolved in a 4:1 DMF:H_2O (6.5 mL) and heated to 125 °C under microwave irradiation for 1 h. Alkyne **S4**, sodium ascorbate (70 mg, 0.35 mmol, 1 Equiv.) and $CuSO_4.5H_2O$ (44 mg, 0.35 mmol, 1 Equiv.) were added to the reaction mixture and left to stir at RT overnight. The reaction mixture was diluted with H_2O (25 mL) and extracted with CH_2Cl_2 (3 × 25 mL), the combined organic extracts were washed with 0.1 M NH_4OH / EDTA $_{(aq)}$ (1 × 25 mL), H_2O (2 × 25 mL) and brine (25 mL) and dried ($MgSO_4$), filtered and concentrated to give a crude material. This crude material was purified by silica gel flash chromatography (20% EtOAc / hexane) to afford the title compound as a white solid (100 mg, 0.32 mmol, 91%).

^{1}H NMR (600 MHz, $CDCl_3$) δ 8.59 – 8.55 (m, $1H_a$), 8.19 (s, $1H_1$), 8.12 (d, *J* = 8.2 Hz, $1H_c$), 7.80 – 7.75 (m, $1H_b$), 4.41 (t, *J* = 7.2 Hz, $2H_2$), 2.45 (t, *J* = 7.1 Hz, $2H_8$), 1.97 – 1.92 (m, $2H_3$), 1.64 – 1.59 (m, $2H_9$), 1.52 – 1.46 (m, $2H_{10}$), 1.39 – 1.27 (m, $6H_{4-6}$), 0.96 (t, *J* = 7.3 Hz, $3H_{11}$), 0.88 (t, *J* = 7.0 Hz, $3H_7$).

^{13}C NMR (151 MHz, $CDCl_3$) δ 151.6, 148.3, 147.7, 139.9, 122.4, 120.4, 119.7, 94.9, 77.5, 50.7, 31.3, 30.8, 30.3, 26.3, 22.6, 22.2, 19.4, 14.1, 13.8.

HRMS-ESI (m/z) Calculated for $C_{19}H_{27}N_4$ [M+H]$^+$, 311.2230; found 311.2226.

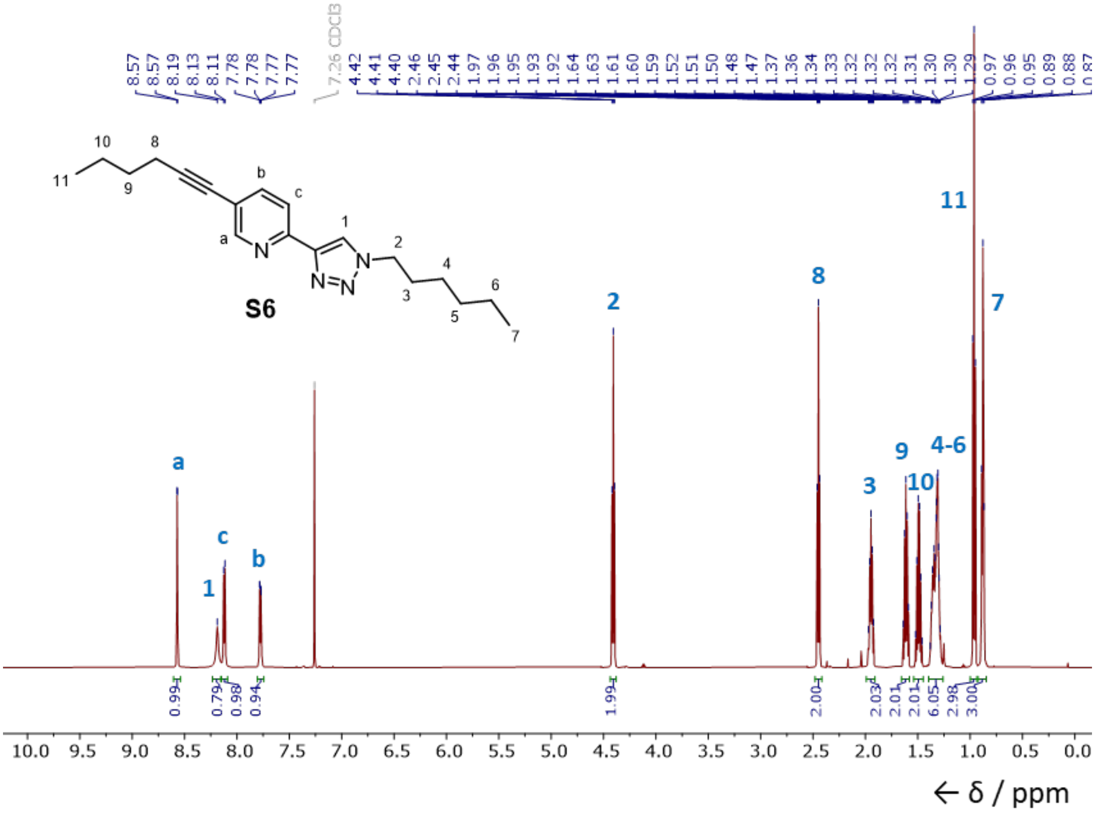

Figure S5. *^{1}H NMR spectrum of compound* ***S6*** *($CDCl_3$, 600 MHz, 298 K).*

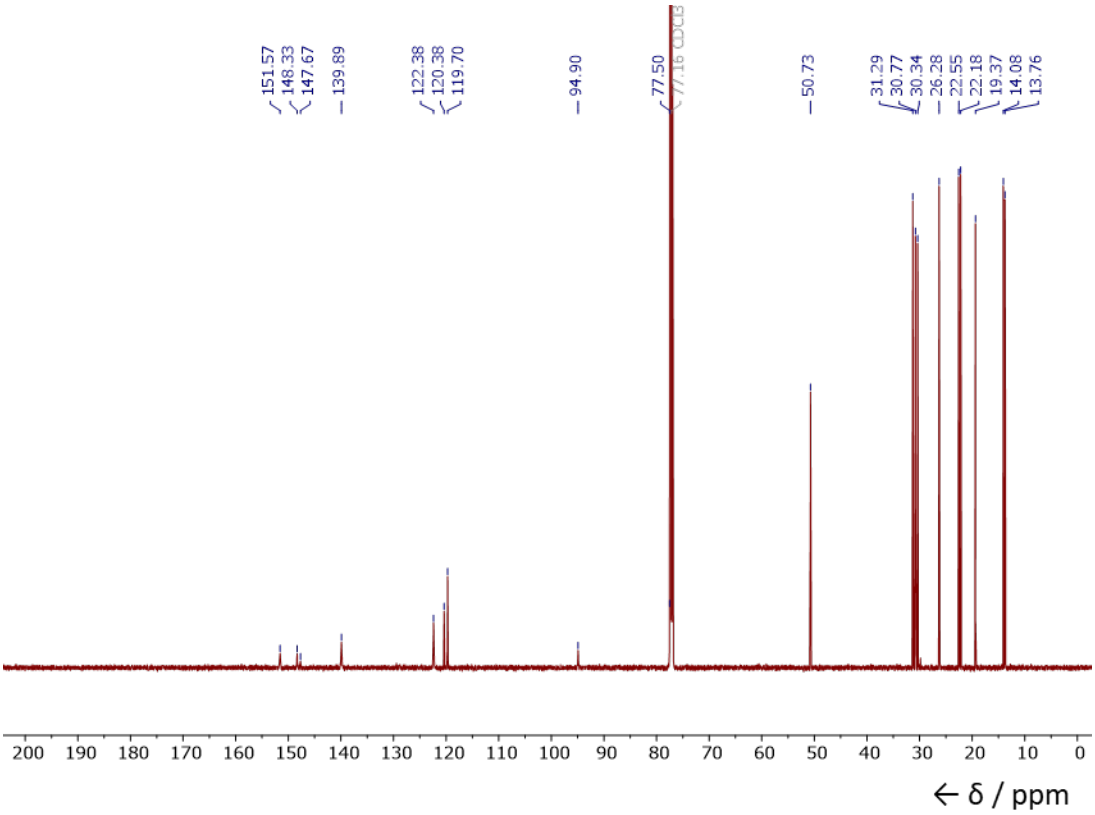

Figure S6. *^{13}C NMR spectrum of compound* ***S6*** *($CDCl_3$, 150 MHz, 298 K).*

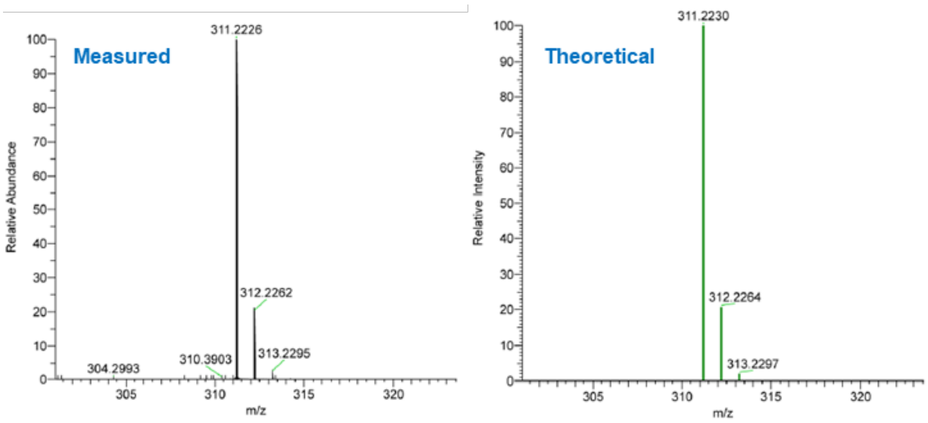

Figure S7. *HRMS spectrum of compound **S6**. Calculated for $C_{19}H_{27}N_4$ $[M+H]^+$, 311.2230; found 311.2226.*

2.7 5-hexyl-2-(1-hexyl-1H-1,2,3-triazol-4-yl)pyridine 1.

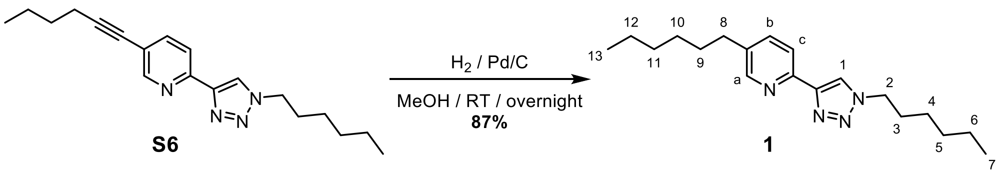

Alkyne **S6** (50 mg, 0.35 mmol, 1 Equiv.) was dissolved in MeOH (10 mL). The reaction mixture was placed under nitrogen, the 10% Pd/C (10 mg) was added. The reaction was stirred under an atmosphere of H_2 at RT overnight. The reaction mixture was filtered through celite and concentrated to afford the title compound as an off white solid (44 mg, 0.14 mmol, 87%).

^{1}H NMR (600 MHz, $CDCl_3$) δ 8.39 (s, $1H_a$), 8.10 – 8.08 (m, $2H_{1\ \&\ c}$), 7.59 (d, *J* = 7.5 Hz, $1H_b$), 4.40 (t, *J* = 7.0 Hz, $2H_2$), 2.63 (t, *J* = 7.4 Hz, $2H_8$), 1.97 – 1.92 (m, $2H_3$), 1.66 – 1.60 (m, $2H_9$), 1.39 – 1.24 (m, $12H_{4\text{-}6\ \&\ 10\text{-}12}$), 0.89 – 0.86 (m, $6H_{7\ \&\ 13}$).

^{13}C NMR (151 MHz, $CDCl_3$) δ 149.5, 148.6, 148.1, 137.5, 136.9, 121.5, 120.0, 50.6, 33.0, 31.8, 31.3, 31.2, 30.4, 28.9, 26.3, 22.7, 22.6, 14.2, 14.1.

HRMS-ESI (m/z) Calculated for $C_{19}H_{31}N_4$ $[M+H]^+$, 315.2543; found 315.2538.

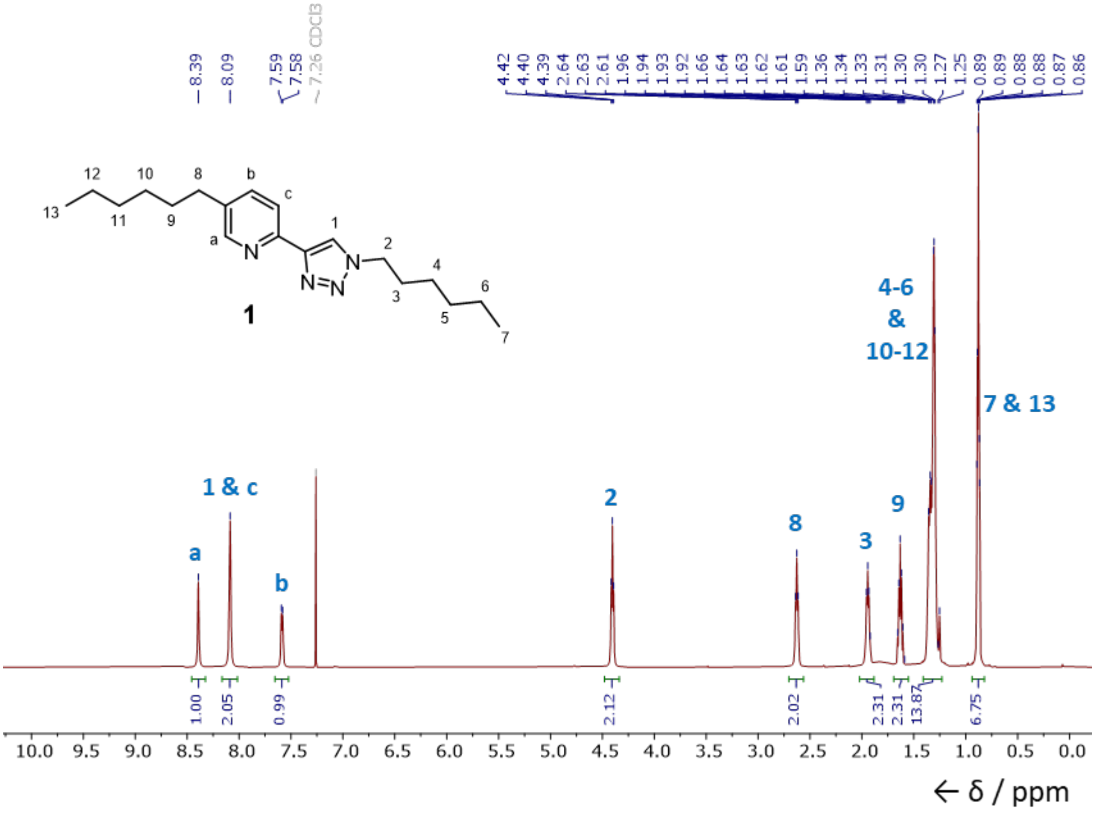

Figure S8. *^{1}H NMR spectrum of compound **1** (CDCl$_3$, 600 MHz, 298 K).*

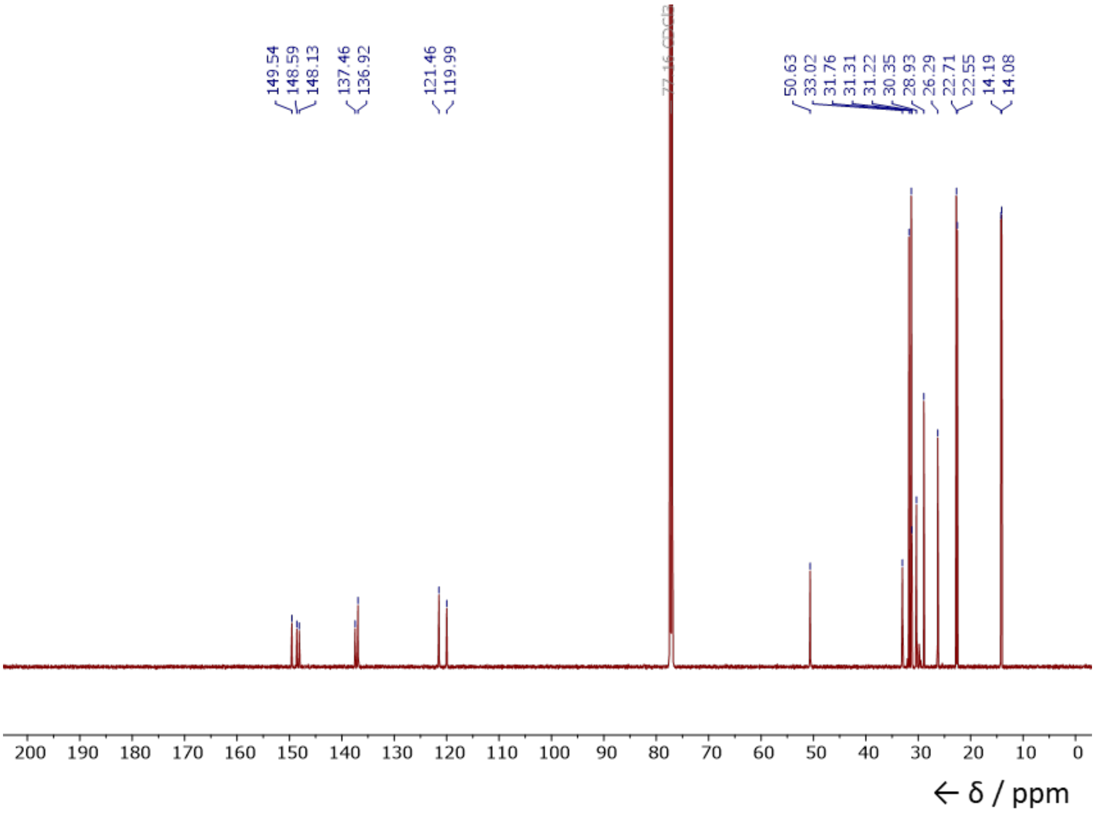

Figure S9. *^{13}C NMR spectrum of compound **1** (CDCl$_3$, 150 MHz, 298 K).*

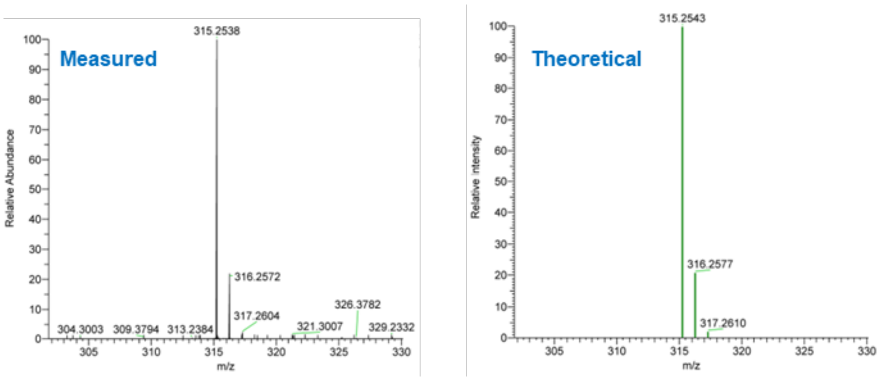

Figure S10. *HRMS spectrum of compound **1**. Calculated for* $C_{19}H_{31}N_4$ *[M+H]*$^+$*, 315.2543; found 315.2538.*

2.8 1-((7-(diethylamino)-3-iodo-2-oxo-2H-chromen-4-yl)methyl)-5-hexyl-2-(1-hexyl-1H-1,2,3-triazol-4-yl)pyridin-1-ium chloride 6:

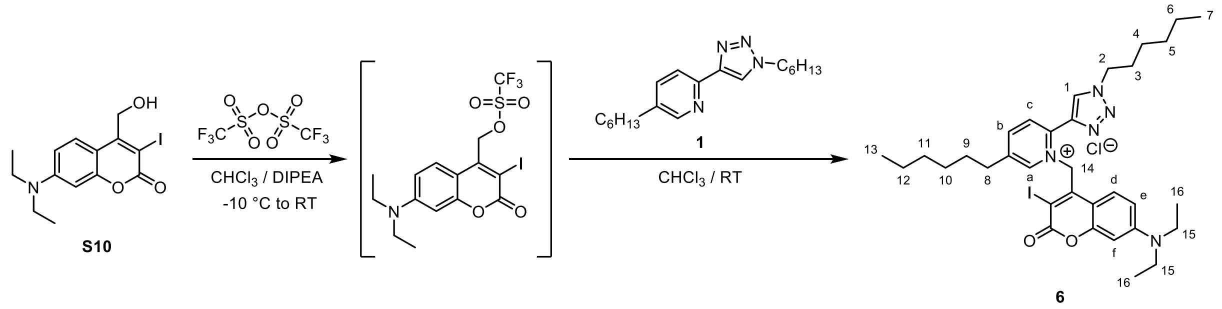

7-(diethylamino)-4-(hydroxymethyl)-3-iodo-2*H*-chromen-2-one **S10** (80 mg, 0.21 mmol, 1.0 Equiv.) was dissolved in anhydrous $CHCl_3$ (15 ml), and *N*,*N*-diisopropylethylamine (0.056 ml, 0.32 mmol, 1.5 Equiv.) was added. Trifluoromethanesulfonic anhydride (40 µl, 0.24 mmol, 1.1 Equiv.) was dissolved in 1 ml of anhydrous $CHCl_3$ at 0 °C and the solution was added to the reaction mixture dropwise at -10 °C. The mixture was stirred for 5 mins, before it was added to a solution of **1** (135 mg, 0.43 mmol, 2.0 Equiv.) in anhydrous $CHCl_3$ (15 ml) at -10 °C. The reaction was slowly warmed to RT and stirred in the dark for 16 hrs before it was quenched with $NaHCO_3$ $_{(sat.\ aq.)}$. The organic phase was then washed with brine (3 × 15 ml) and dried ($MgSO_4$). The solvent was removed under reduced pressure to afford the crude product. The crude product was purified through silica gel flash chromatography (5% - 10% MeOH/$CHCl_3$) to give the title compound as a brown solid (30 mg, 0.21 mmol, 21%).

^{1}H NMR (400 MHz, $CDCl_3$) δ 11.47 (s, $1H_1$), 8.67 – 8.53 (m, $2H_{a\ \&\ c}$), 7.86 (d, *J* = 6.4 Hz, $1H_b$), 7.26 (d, *J* = 8.8 Hz, $1H_d$), 6.71 (s, $2H_{14}$), 6.52 (d, *J* = 2.0 Hz,$1H_f$), 6.45 (d, *J* = 8.3 Hz, $1H_e$), 4.71 (t, *J* = 7.0 Hz, $2H_2$), 3.39 (q, *J* = 7.1 Hz, $4H_{15}$), 2.73 (t, *J* = 7.6 Hz, $2H_8$), 2.11-1.95 (m, $2H_3$), 1.74 – 1.62 (m, $2H_9$), 1.39 – 1.13 (m, $18H_{4\text{-}6\ \&\ 10\text{-}12\ \&\ 16}$), 0.93 – 0.76 (m, $6H_{7\ \&\ 13}$).

^{13}C NMR (101 MHz, $CDCl_3$) δ 158.4, 155.8, 151.4, 149.7, 148.7, 141.6, 140.5, 140.4, 138.7, 132.0, 126.3, 125.1, 109.8, 107.1, 97.4, 88.1, 58.6, 54.7, 44.9, 33.1, 31.5, 30.8, 30.7, 29.4, 28.8, 25.6, 22.5, 22.3, 14.1, 13.9, 12.4.

HRMS-ESI (m/z) Calculated for $C_{33}H_{45}IN_5O_2^+$ [M]$^+$, 670.2613; found 670.2623.

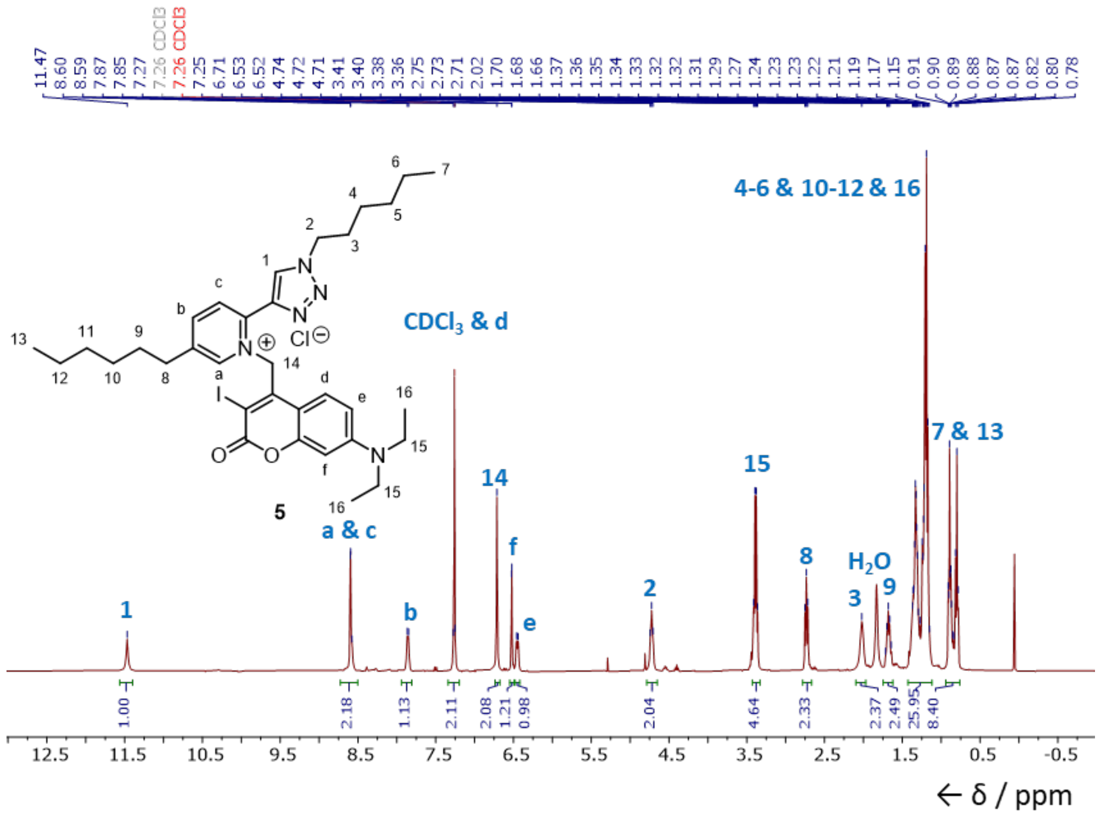

Figure S11. *^{1}H NMR spectrum of compound **6** ($CDCl_3$, 400 MHz, 298 K).*

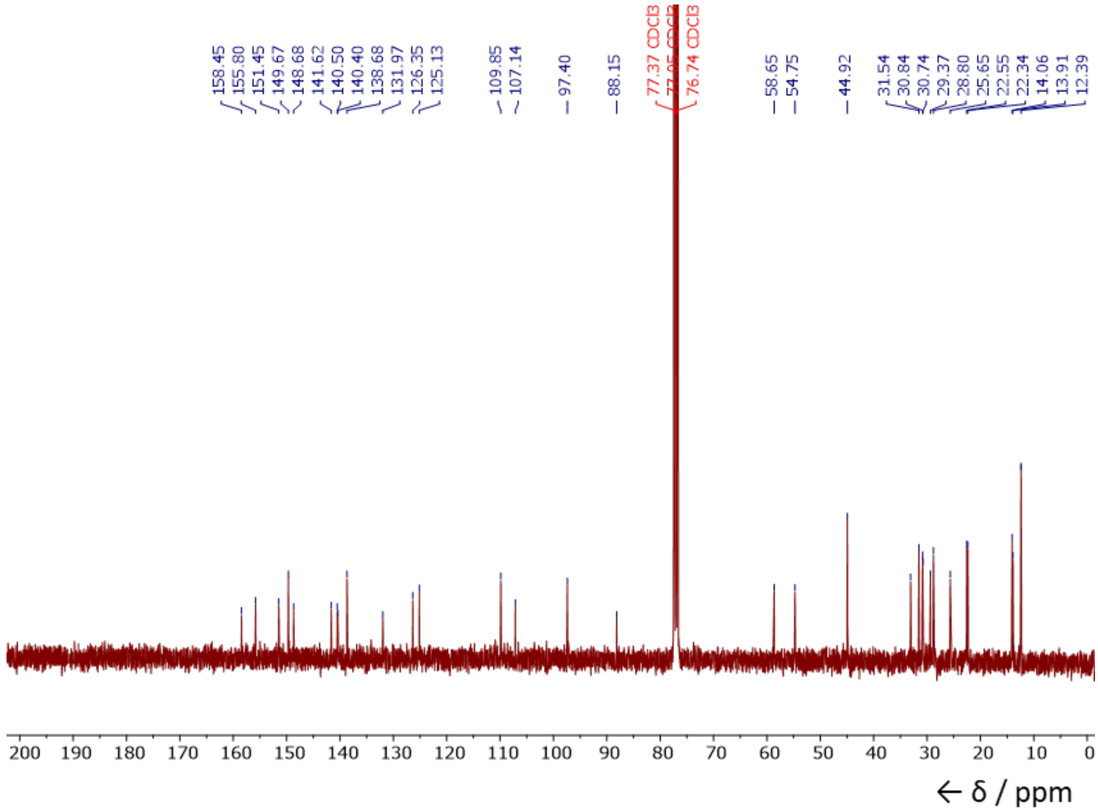

Figure S12. *^{13}C NMR spectrum of compound **6** ($CDCl_3$, 101 MHz, 298 K).*

Expanded Spectrum RT 0.12, NL 4698335, Peak [1], Target Mass 670.2613

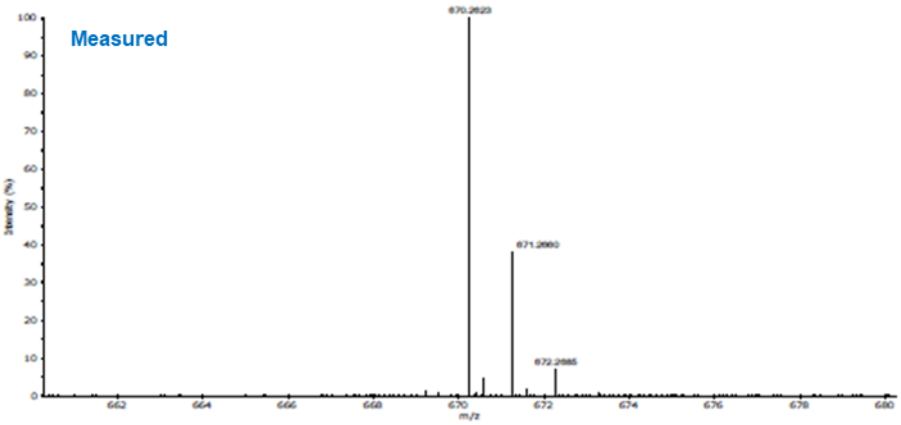

Theoretical Spectrum for C33H45IN5O2, Minimum Abundance 0.01%

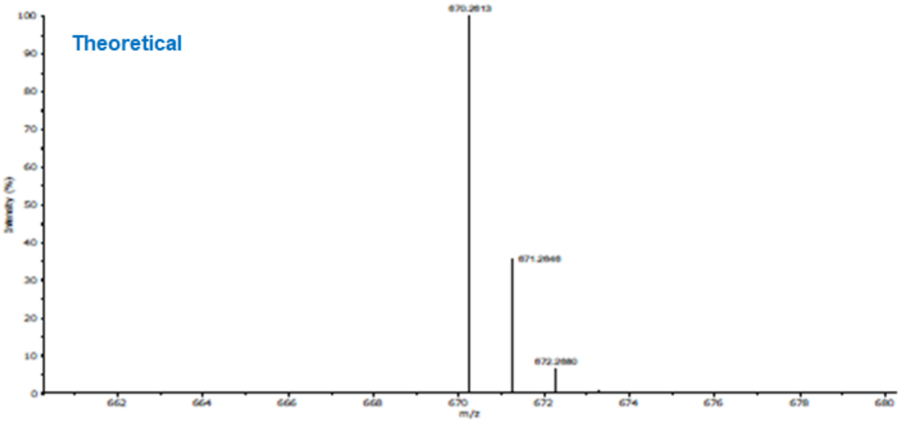

Figure S13. *HRMS spectrum of compound **6**. Calculated for* $C_{33}H_{45}IN_5O_2^+$ *[M]*$^+$*, 670.2613; found 670.2623.*

3 UV-Visible absorption analysis

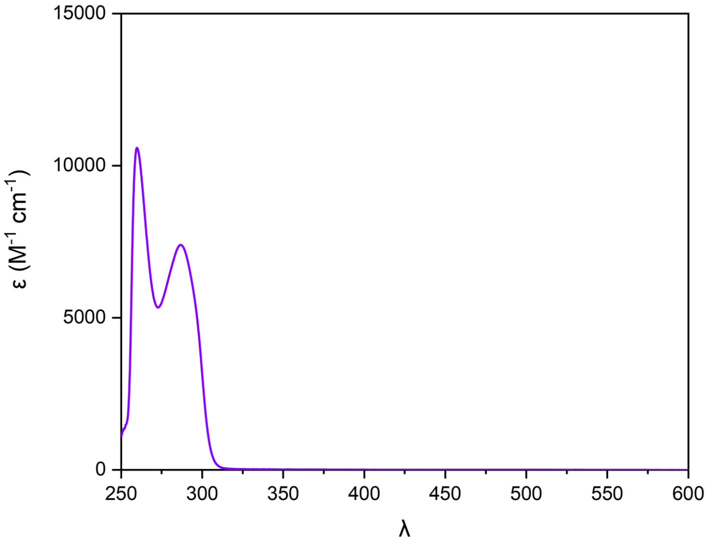

Figure S14. *UV-vis spectra of **1** in DMSO.*

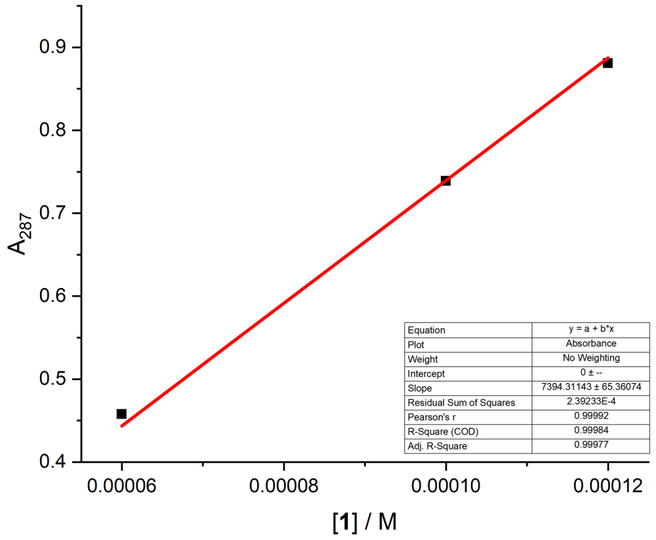

Figure S15. *Beer-Lambert plots for **1** at 287 nm.*

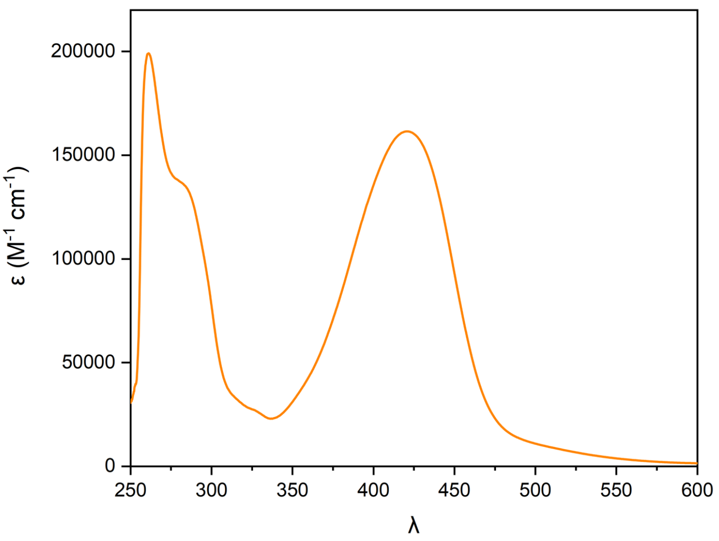

Figure S16. *UV-vis spectra of **6** in DMSO.*

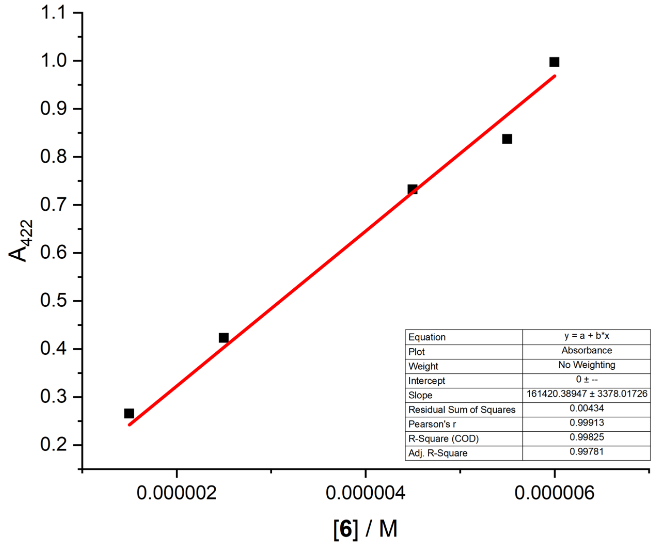

Figure S17. *Beer-Lambert plots for **6** at 287 nm.*

4 Pd(II) transport experiments

4.1 Vesicle preparation

A thin film of lipid was formed by evaporating a chloroform solution under reduced pressure on a rotary evaporator (25 °C) and then under high vacuum for 6 hours. The lipid film was hydrated by vortexing with the prepared buffer (100 mM $NaNO_3$, 10 mM HEPES, 2 mM TPPTS, 1 mM 8-(allyloxy)pyrene-1,3,6-trisulfonic acid trisodium salt (allyl-HPTS **4**), pH 7.0). The lipid suspension was then subjected to 5 freeze-thaw cycles using liquid nitrogen and a water bath (40°C) followed by extrusion 19 times through a polycarbonate membrane (pore size 200 nm). Extra-vesicular components were removed by size exclusion chromatography on a Sephadex G-25 column eluted with 100 mM $NaNO_3$, 10 mM HEPES, pH 7.0. Final conditions: LUVs (2.5 mM lipid); inside 100 mM $NaNO_3$, 10 mM HEPES, 2 mM TPPTS, 1 mM allyl-HPTS **4**, pH 7.0; outside: 100 mM $NaNO_3$, 10 mM HEPES, pH 7.0.

4.2 Transport assays with Allyl-HPTS

In a typical experiment, the LUVs containing allyl-HPTS (120 µL, final lipid concentration 100 µM) and the test transporter (various concentrations, in 7.5 µL DMSO) were added to buffer (2880 µL of 100 mM $NaNO_3$, 10 mM HEPES, pH 7.0) at 25°C under gentle stirring. The experiment was started and a pulse of $Pd(NO_3)_2$ (30 µL of 10 mM solution in water, final concentration 100 µM) was added immediately. The fluorescence emission was monitored at λ_{em} = 510 nm (λ_{ex} = 460 nm).

Data was normalised using equation S1.

$$I_{rel} = \frac{I_t - I_0}{I_{max} - I_{min}} \qquad \textbf{(S1)}$$

where I_t is the fluorescence intensity at time t, I_0 is the fluorescence intensity at time 0 when the Pd^{2+} gradient is established, I_{max} is the fluorescence intensity of 1.0 mol% of **1** (w.r.t lipid) after 1000 s, and I_{min} is the fluorescence intensity of 1.0 mol% of **1** (w.r.t lipid) at 0 s. Each individual concentration was repeated at least twice and averaged; error bars represent standard deviations.

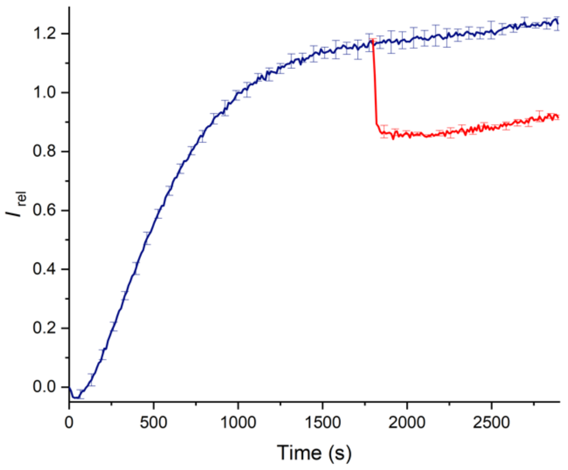

Figure S18. *Ion transport allyl-HPTS assay data for* ***1*** *DMSO, 1 mol% to lipid) (blue) and control with second addition of Pd(NO_3)$_2$ (30 µL of 10 mM solution in water) (red). Absence of further reaction indicted by identical gradient after Pd(II) addition.*

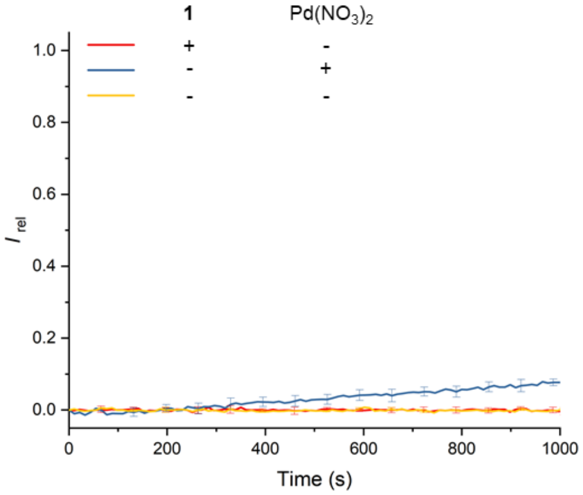

Figure S19. *Ion transport allyl-HPTS assay control data for* ***1*** *(1 mol% to lipid): blank with no Pd(NO_3)$_2$ (red), blank with 100 µM Pd(NO_3)$_2$ but no transporter (blue), and blank with only water addition (yellow).*

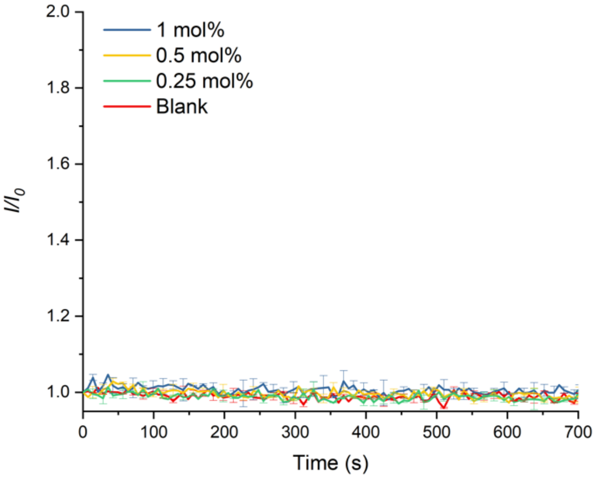

Figure S20. *Ion transport allyl-HPTS assay data in the absence of TPPTS for* ***1*** *(0-1 mol% to lipid) in POPC LUVs.*

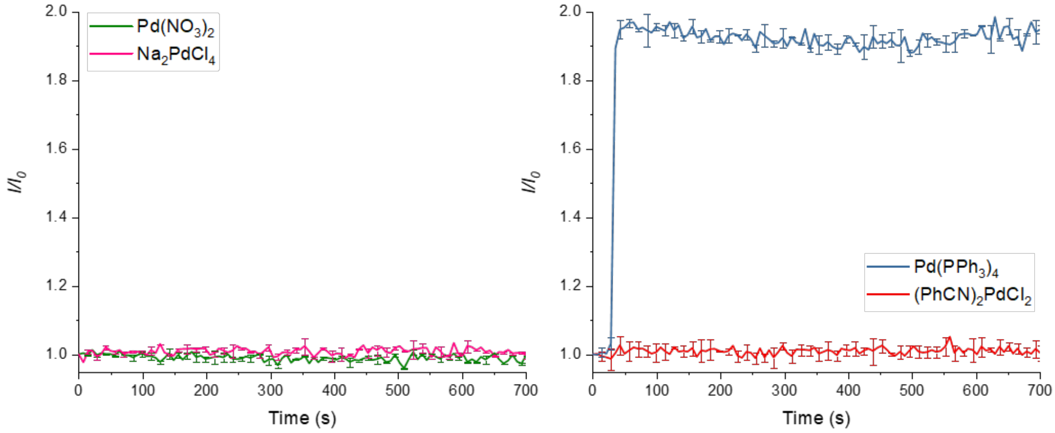

Figure S21. *Control experiments for the allyl-HPTS assay in the absence of TPPTS for different palladium sources added at 30 s.* ***Left****, hydrophilic Pd^{II} salts (10 mM in H_2O, final concentration 100 µM) and* ***right****, lipophilic complexes of Pd^0 or Pd^{II} (10 mM in THF, final concentration 100 µM) added to POPC LUVs.*

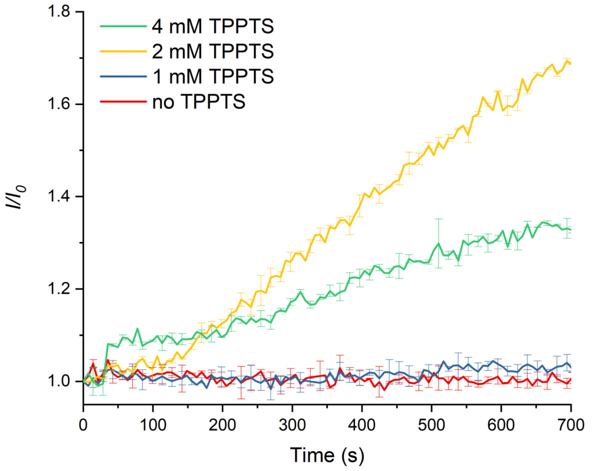

Figure S22. *Ion transport allyl-HPTS assay data for* ***1*** *(1 mol% to lipid) with different concentrations of TPPTS (0-4 mM) encapsulated within the POPC LUVs.*

5 Pd(II) catalysis experiments in solution

To an aqueous solution of 10 µM allyl-HPTS **4** (1980 µL, 100 mM $NaNO_3$, 10 mM HEPES, pH 7.0 with various concentrations of TPPTS) $Pd(NO_3)_2$ (20 µL of 10 mM solution in water, final concentration 100 µM) was added after 60 s.

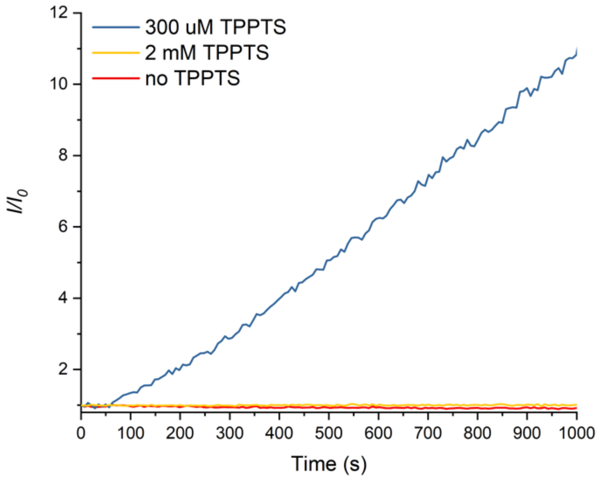

Figure S23. *Change in normalised fluorescence upon addition of $Pd(NO_3)_2$ (final concentration 100 µM) to a solution of **4** in $NaNO_3$ buffer with various concentration of TPPTS (0-2 mM) present.*

6 Photo-deprotection experiments

Photo-deprotection of **6** was conducted in the DMSO-d_6 solution by irradiating with Thorlabs mounted LEDs at 455 nm (L455M3, 1W) in a Thorlabs cuvette holder (CVH100/MM). The LEDs were supplied with 1 A current using Thorlabs T-cub LED driver (LEDD1B). The deprotection was monitored using ^{1}H NMR experiments at known time intervals. The percentage deprotection was then calculated using Mestrenova software to integrate peaks corresponding to compound **1** and the photo-caged **6** using equation S2.

$$\%deprotection = \frac{I_{free}}{I_{caged} + I_{free}} \qquad \textbf{(S2)}$$

where I_{free} and I_{caged} are integrals of analogous protons on **1** and **6**, respectively.

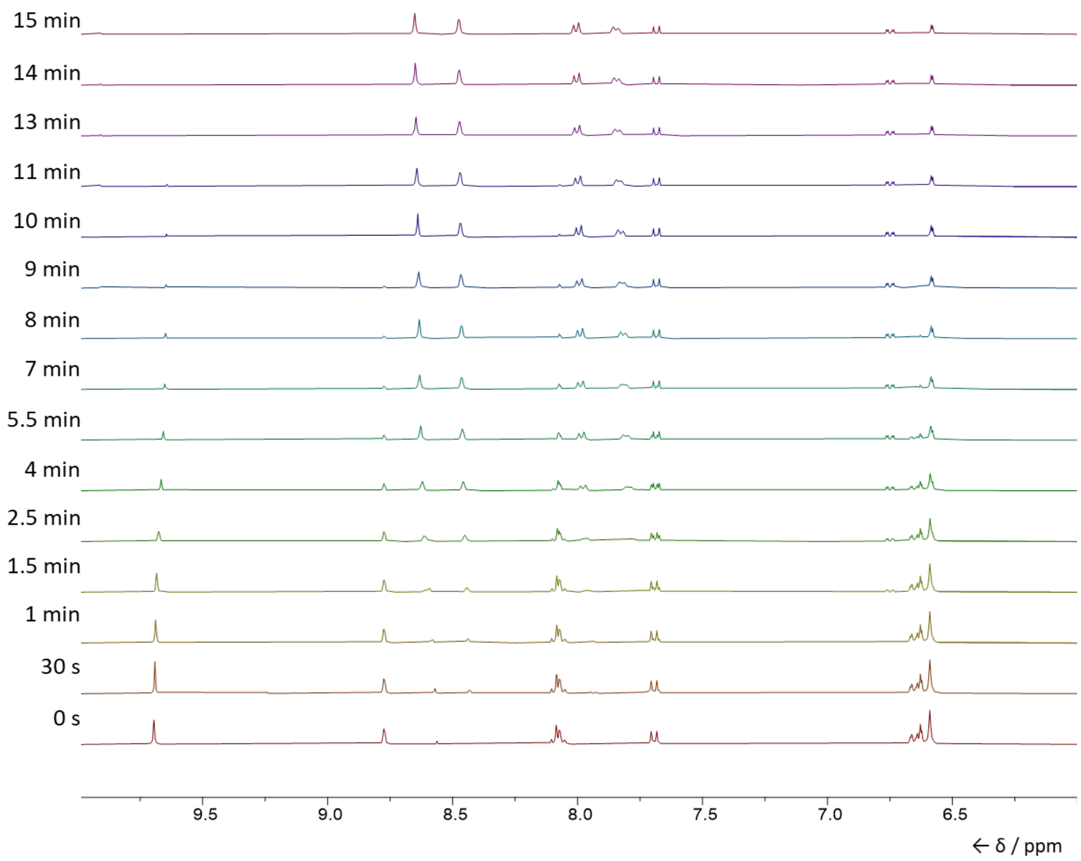

Figure S24. *The ^{1}H NMR spectra of compound **6** in DMSO-d_6 at 298 K, after given irradiation times with 455 nm light.*

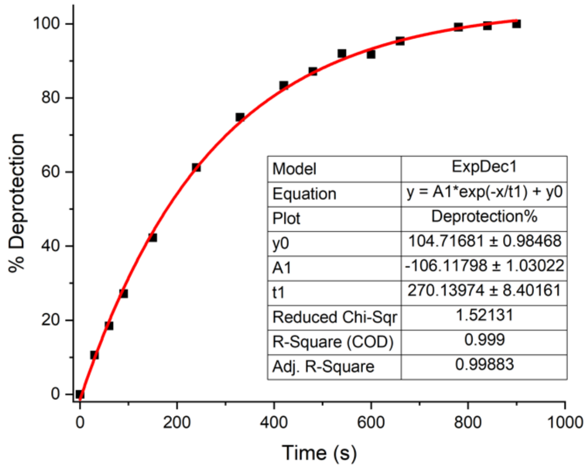

Figure S25. *The % deprotection of* ***6*** *after irradiation with a 455 nm LED, with a half-life of 187 s.*

7 Irradiation studies on Pd(II) transport assays

In *ex-situ* irradiation experiments, a 400 µM solution of **6** in DMSO was irradiated for 1 hr with a 455 nm LED. Caged transporter **6** (irradiated or non-irradiated, 400 µM, in 7.5 µL DMSO, 1 mol% to lipid) and the LUVs containing allyl-HPTS (120 µL, final lipid concentration 100 µM) were added to buffer (2880 µL of 100 mM $NaNO_3$, 10 mM HEPES, pH 7.0) at 25°C. The transport experiment was then conducted as described in section **4.2**.

In *in-situ* irradiation experiments, **6** (400 µM, in 7.5 µL DMSO, 1 mol% to lipid) and the LUVs containing allyl-HPTS (120 µL, final lipid concentration 100 µM) were added to buffer (2880 µL of 100 mM $NaNO_3$, 10 mM HEPES, pH 7.0) at 25°C. The system was irradiated with 455 nm light for the given period of time. The transport experiment was then conducted as described in section **4.2**.

8 Calcein leakage assays

POPC vesicles were prepared containing a high concentration of calcein sufficient to cause self-quenching of the emission, in similar procedures stated in **4.1**. Final conditions: LUVs (2.5 mM lipid); inside 100 mM $NaNO_3$, 10 mM HEPES, 100 mM calcein, pH 7.0; outside: 100 mM $NaNO_3$, 10 mM HEPES, pH 7.0.

In a typical experiment, the LUVs containing calcein (120 µL, final lipid concentration 100 µM) and the test transporter (7.5 µL of 400 µM solution in DMSO, 1 mol% to lipid) were added to buffer (2880 µL of 100 mM $NaNO_3$, 10 mM HEPES, pH 7.0) at 25°C under gentle stirring. The experiment was started and a pulse of $Pd(NO_3)_2$ (30 µL of 10 mM solution in water, final concentration 100 µM) was added immediately. The fluorescence emission was monitored at λ_{em} = 520 nm (λ_{ex} = 490 nm). The assay was calibrated at 1000 s with detergent (40 µL of 11% Triton X-100 in 7:1 (v/v) H_2O-DMSO).

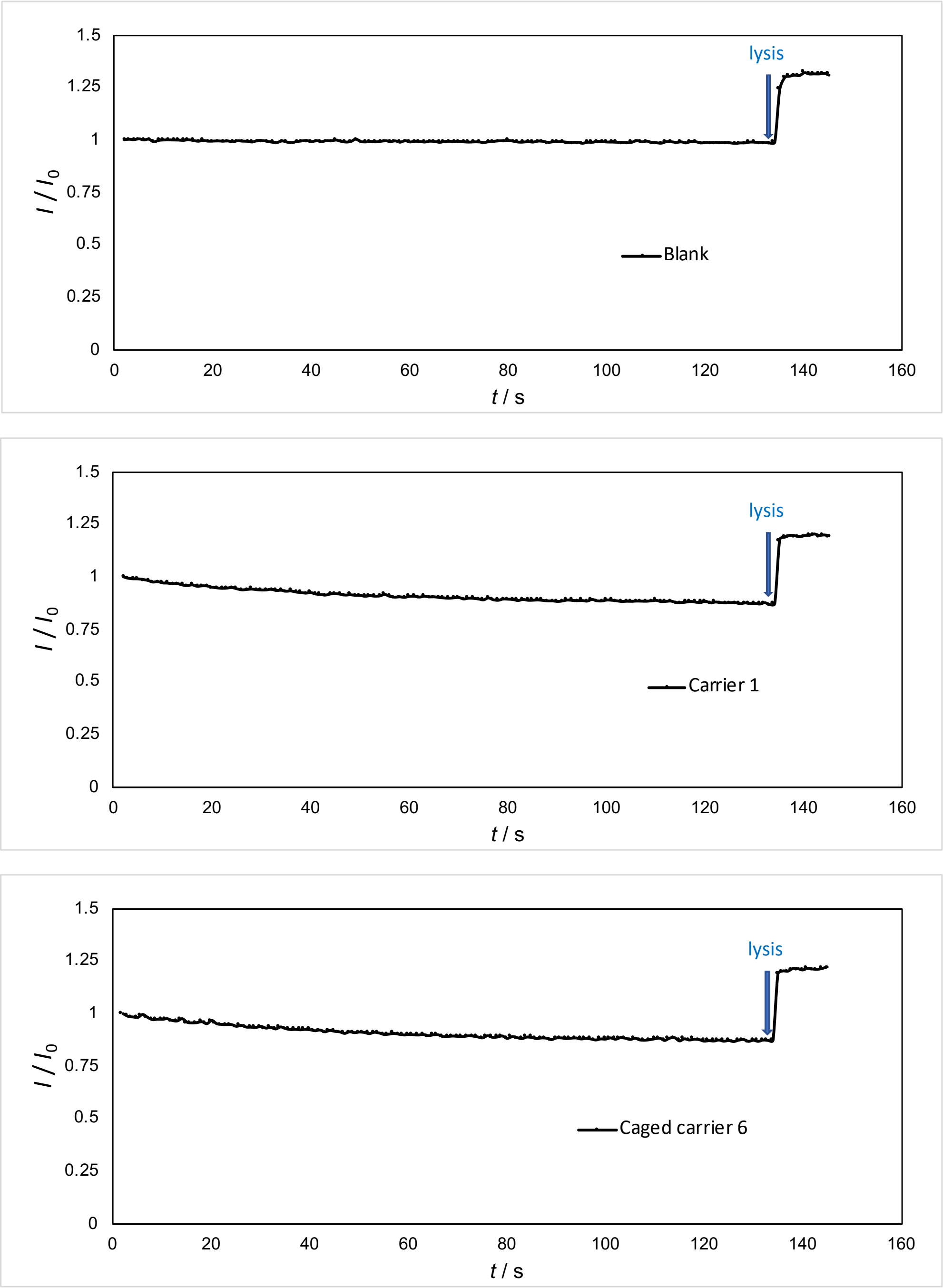

Figure S26. Calcein release assay with DMSO blank, **1** and **6** (1 mol% with respect to lipid). Data represents the average of 3 repeats. Data for carrier **6** is after photoirradiation for 1 hr at 455 nm (conditions as in Fig. 4B). Arrow indicates addition of Triton X-100 to lyse the LUVs.

9 Dynamic light scattering

Table S1. Dynamic light scattering (DLS) LUV size data (conditions as in section 4.2 for catalysis experiments, with external 100 µM $Pd(NO_3)_2$), and in the presence of **1** and **6** (1 mol% with respect to lipid). Data for carrier **6** is after photoirradiation for 1 hr at 455 nm (conditions as in Fig. 4B).

	Average diameter / nm	PDI
LUVs + Pd(II) + **1**	117	0.55
LUVs + Pd(II) + **6**	105	0.61
LUVs + Pd(II)	98	0.65

10 References

1. J. T. Fletcher, B. J. Bumgarner, N. D. Engels and D. A. Skoglund, *Organometallics*, 2008, **27**, 5430-5433.
2. T.-H. Fu, Y. Li, H. D. Thaker, R. W. Scott and G. N. Tew, *ACS Med. Chem. Lett.*, 2013, **4**, 841-845.
3. G. Liu, M. Xue and Y. Zhou, *Russ. J. Appl. Chem.*, 2016, **89**, 1861-1868.
4. C. Bianchini, G. Giambastiani, I. G. Rios, A. Meli, W. Oberhauser, L. Sorace and A. Toti, *Organometallics*, 2007, **26**, 5066-5078.
5. Y. Takayama, T. Hanazawa, T. Andou, K. Muraoka, H. Ohtani, M. Takahashi and F. Sato, *Org. Lett.*, 2004, **6**, 4253-4256.
6. J. Kaufmann, P. Müller, E. Andreadou and A. Heckel, *Chem. Eur. J.*, 2022, **28**, e202200477.
7. M. Klausen, V. Dubois, G. Clermont, C. Tonnele, F. Castet and M. Blanchard-Desce, *Chem Sci*, 2019, **10**, 4209-4219.
